# β‐Triketone‐Based Ionizable Cationic Lipids Synthesized via Click Chemistry for siRNA Delivery

**DOI:** 10.1002/advs.202515482

**Published:** 2026-03-30

**Authors:** Huatian Li, Haocheng Tang, Yiqing Mu, Shangyu Chen, Paul Edward Floreancig, Junmei Wang, Yixian Huang, Song Li

**Affiliations:** ^1^ Center for Pharmacogenetics Department of Pharmaceutical Sciences School of Pharmacy University of Pittsburgh Pittsburgh Pennsylvania USA; ^2^ Computational Chemical Genomics Screening Center Department of Pharmaceutical Sciences School of Pharmacy University of Pittsburgh Pittsburgh Pennsylvania USA; ^3^ Department of Chemistry Dietrich School of Arts and Sciences University of Pittsburgh Pittsburgh Pennsylvania USA

**Keywords:** click chemistry, ionizable lipids, lipid nanoparticles, SiRNA, β‐triketone

## Abstract

Lipid nanoparticles (LNPs) have demonstrated their effectiveness as carriers for the delivery of RNA therapeutics. As the core components of the LNP system, ionizable cationic lipids (ICLs) are still the major focus of research in this field. In this study, we reported a class of novel β‐triketone‐based ICLs synthesized via click chemistry. We demonstrated that the reaction could be completed instantaneously, and a library of ICLs was generated in a short time. In vitro and in vivo screening identified a lipid that was comparable to FDA approved Dlin‐MC3‐DMA lipid in siRNA LNPs‐mediated gene silencing. In addition, we adopted a computational approach to characterizing the click chemistry in lipid synthesis, as well as the binding between siRNA and ICLs. Our work offers a novel approach to synthesizing ICLs of new structural features, which may not only improve our understanding of the structure‐activity relationship (SAR) of ICLs but also lead to the development of improved LNPs for more effective delivery of nucleic acid therapeutics.

## Graphical abstract

This study innovatively applies β‐triketone‐based click chemistry in the synthesis of ionizable cationic lipids (ICLs), which allows the generation of a lipid library in large quantities and in a short time. The ICLs identified achieved effective lipid nanoparticles‐mediated hepatic gene silencing in vivo using siRNA, and computational simulations are completed to study the characteristics of the synthesized ICLs to provide further knowledge for the development of improved LNPs.

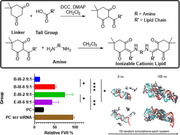

## 1 Introduction

LNPs have demonstrated their incredible potential as effective carriers for the delivery of RNA therapeutics by overcoming the formidable physiological and biological barriers [1]. ICLs are the key component in LNPs, but the rational design of these lipids is still hindered by a lack of sufficient knowledge about their structure‐activity relationship (SAR) [2, 3]. Current research focuses on generating a large number of new lipids for further SAR study and identification of an optimal ICL for a particular application [4]. In addition to the efficiency of delivery, another important aspect of the lipids is the simplicity of synthesis and the feasibility of scale‐up, which is critical for the translation to the clinic [5].

**FIGURE 1:**
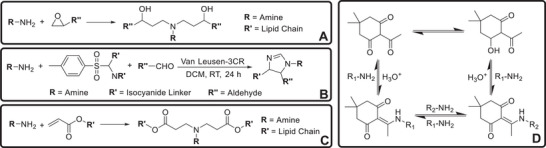
Existing strategies for synthesis of ICLs via (A) ring‐opening reactions of epoxides, (B) Van Leusen reaction and (C) Michael addition reactions. (D) Synthesis of recyclable plastics by Helms et al.

Several strategies have been reported for the synthesis of ICLs, such as ring‐opening reactions of epoxides, Van Leusen reaction, and Michael addition reaction (Figure 1A–C) [6, 7, 8, 9]. In this study, we explored the potential of β‐triketones in pharmaceutical and biomedical applications inspired by Helms’ seminal work on their application in recyclable plastics (Figure 1D) [10, 11, 12, 13]. Herein, we show that ICLs can be synthesized via click condensation reaction between β‐triketones and amines [14]. We first characterized the efficiency and selectivity of this chemistry. A small library of 50 ICLs were then synthesized and evaluated for the efficiency in siRNA LNPs‐mediated gene knockdown in vitro and in vivo. Finally, we introduced a computational approach to define the lipid/siRNA interactions and their impact on siRNA delivery efficiency.

## 2 Results and Discussion

### 2.1 Synthesis of ICL Library

**FIGURE 2:**
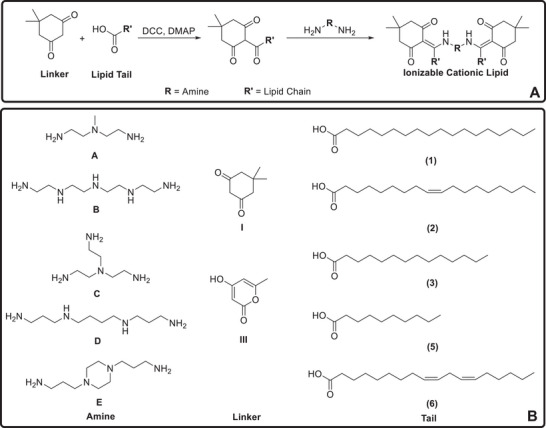
(A) Synthesis of ICLs using β‐diketone linker. (B) Scope of the current ICL library.

A typical ICL has three motifs: an amine head, a linker, and a lipid tail [15]. In this library, five amine heads, two β‐diketones, and five lipid tails were selected to synthesize a total of 50 lipids of various structures (Figure 2). The amine heads featured tertiary amines, secondary amines or 1,4‐diazinane ring, and differed in the number of amines and “arms” that allow for conjugation with lipid tails. The lipid tails varied in the degree of unsaturation and length of carbon chain. The synthesis of lipids could be completed with only two steps of reaction. The first step is to conjugate the linker and tail with the help of a condensing agent to form tri‐ketone lipids. The second step is to mix di/tri‐amine with tri‐ketone lipid to form an imine structure, and the reaction could be completed within 5 min.

### 2.2 β‐Triketone‐Based Click Chemistry

**FIGURE 3:**
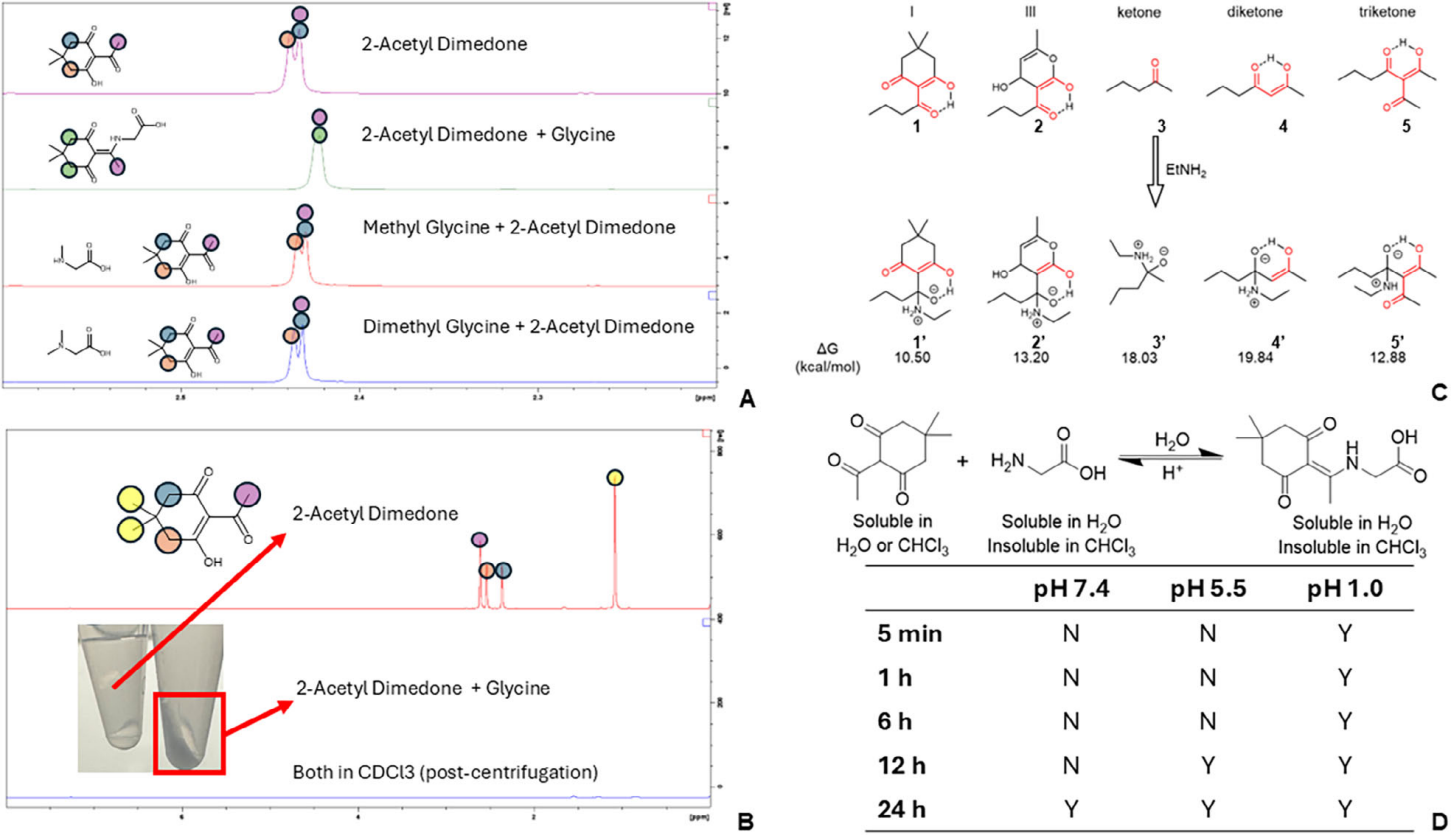
(A) Regional ^1^H‐NMR spectra of 2‐acetyl dimedone and its post‐reaction product with glycine, methyl glycine, and dimethyl glycine in D_2_O showing the α‐hydrogens in β, β’‐triketone‐glycine. (B) Full ^1^H‐NMR spectra of 2‐acetyl dimedone and its post‐reaction product with glycine in the solvent and solid phases following CDCl_3_ extraction. (C) Calculated Gibbs free energy differences among the model β, β’‐triketone‐lipids of linker I (1) and III (3), linear mono‐ (3), di‐ (4), tri‐ketone (5), and their respective intermediate of addition reaction with ethylamine (1’‐5’). (D) Mechanism of β, β’‐triketone‐glycine degradation and a summary table of its pH‐dependent degradation. Y = Detection of 2‐acetyl dimedone in extraction supernatant, N = No detection of 2‐acetyl dimedone in extraction supernatant.

To demonstrate the click chemistry nature of this reaction, glycine and its derivatives with different types of amines were selected to characterize the reactions. Glycine contains one primary amine, while methyl glycine and dimethyl glycine each contain one secondary or tertiary amine, respectively. The 2‐acetyl dimedone was used to react with amines in 1:1 molar ratio and demonstrate the completion of reaction. Both reactants were mixed in H_2_O, and 5 min later, the mixture was frozen and lyophilized prior to re‐dissolving in D_2_O to obtain ^1^H‐NMR spectra (Figure 3A; Figure S1). The changes of peaks corresponding to the protons in 2‐acetyl dimedone marked the completion of its reaction with the primary amine in glycine. Specifically, the peak representing the enolic proton of the β‐triketone moiety that was predominantly present in the enol tautomer prior to the reaction disappeared and merged with its ketonic proton after the reaction, indicating that both protons at α positions have become chemically equivalent [16, 17]. However, the peak representing the enolic proton of the β‐triketone did not change after mixing methyl glycine or dimethyl glycine with 2‐acetyl dimedone, suggesting that the reaction did not take place. This showed that the reaction could be completed in an aqueous environment almost instantaneously (≤ 5 min), with high selectivity toward the primary amine. 2‐Acetyl dimedone is soluble in both water and chloroform, while glycine and the conjugate are only soluble in water. CDCl_3_ extraction of lyophilized reaction mixture showed obvious precipitation with no 2‐acetyl dimedone found in the extraction solvent (Figure 3B), further confirming the fast completion of the reaction. Similarly, CDCl_3_ extraction was used to study the degradation of glycine‐2‐acetyl dimedone conjugate (Figure 3D). The completed reaction mixture was adjusted to pH 7.4, 5.5, and 1.0, and samples taken at different timepoints were lyophilized and extracted with CDCl_3_. Under pH 7.4, 2‐acetyl dimedone could not be detected by ^1^H‐NMR in CDCl_3_ until 24 h, in contrast to 12 h under pH 5.5, and 5 min under pH 1.0. This result showed the pH‐dependent degradation of β, β’‐triketone bond (Figure 3D; Figures S2–S4). ICLs with β, β’‐triketone linkage are likely to be stable in blood but undergo degradation in the acidic endosomal‐lysosomal compartments following intracellular delivery.

### 2.3 Simulated Activation Energy in Schiff Base Formation

We also used a computational approach to characterize this reaction by comparison to the formation of other linear Schiff bases (Figure 2C). Under neutral or mildly acidic conditions, Schiff base formation is typically rate‐limited by nucleophilic addition of an amine to a ketone carbonyl [18]. To understand the reactivity of different linker scaffolds used for siRNA complexation, we modeled the rate‐determining step for Linkers I (1) and III (2) together with simpler analogs: mono‐ketone (3), linear diketone (4), and linear triketone (5). Activation energies were estimated using reaction enthalpies via the Evans–Polanyi relationship, appropriate for structurally similar systems undergoing homologous reactions [19, 20]. Notably, Linker III (2) exhibited a higher activation barrier than Linker I (1), mainly due to fewer carbonyl groups in its structure. This trend corresponds to that observed for linear ketones, where introducing an additional carbonyl group (from di‐ to tri‐ketone) lowers the activation energy by stabilizing the transition state and reducing the penalty from conjugation loss, as shown by comparison between (4) and (5). The elevated barrier in Linker III reflects the absence of such stabilization, suggesting slower degradation—a property that may enhance in vivo siRNA delivery by prolonging linker integrity. Furthermore, the cyclic architecture of both linkers plays a critical role. Compared to their linear counterparts, both linkers I and III benefit from preorganized, rigid conformations that promote favorable intramolecular interactions in the transition state. The introduction of cyclic ketones can enhance reaction selectivity, enabling the Schiff base reaction to occur specifically on the ketones within the lipid chains [21].

### 2.4 In Vitro Characterizations of ICLs‐Mediated Transfection

**FIGURE 4:**
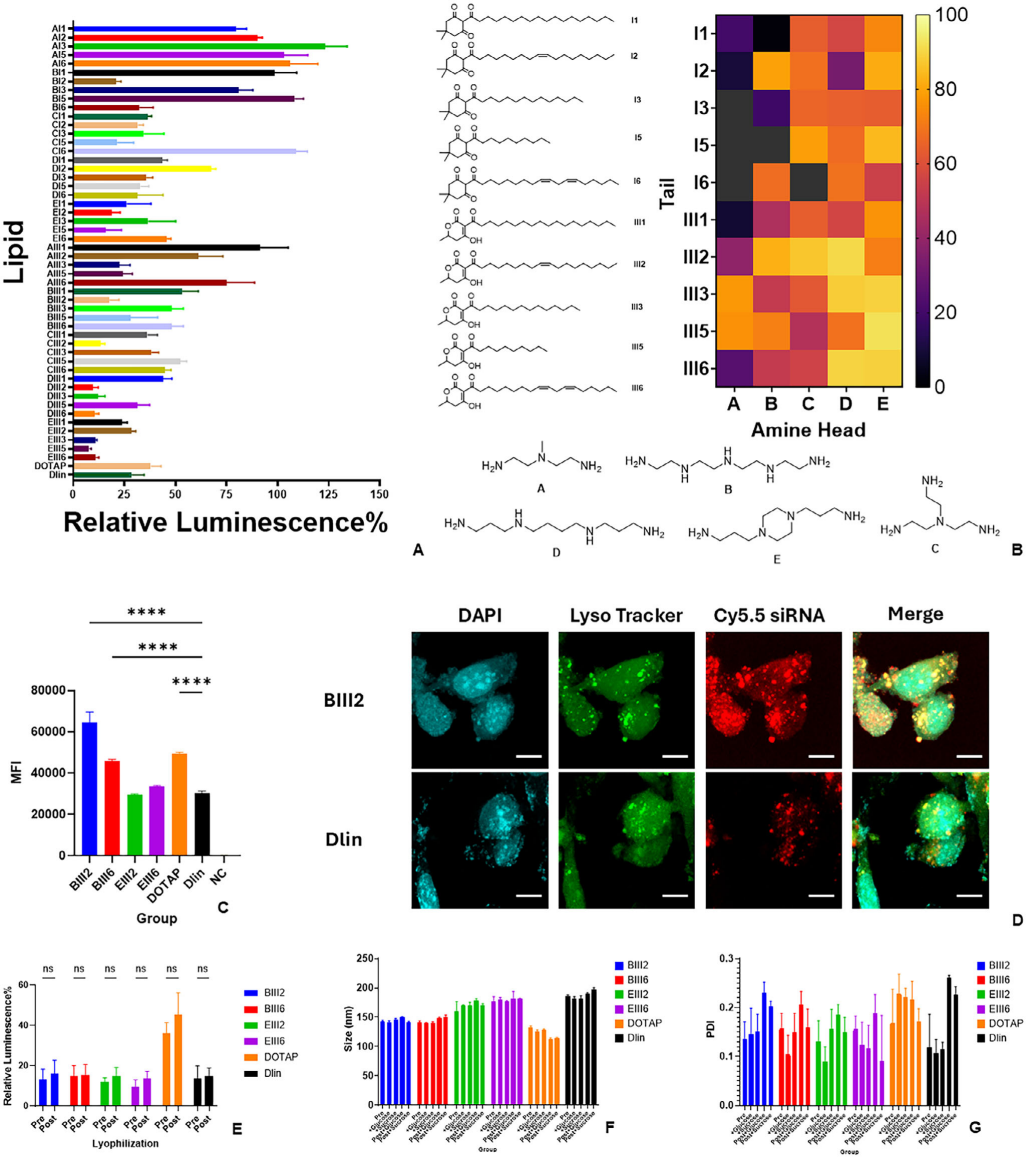
(A) Relative luminescence of luciferase‐expressing HepG2 cells following treatment of LNPs in comparison to cells receiving no treatment (1 µg siRNA, *n* = 3). (B) Heatmap analysis of gene silencing efficiency of synthesized ionizable cationic lipids and their structural features. (C) Cellular uptake of LNPs in HepG2 cells determined by flowcytometry (*n* = 3). (D) Confocal fluorescence microscopic images of HepG2 cells at 4 h post‐treatment with Cy5.5‐siRNA encapsulated LNPs. (Scale bar: 10 µm). (E) Gene‐silencing activity of LNPs before and after lyophilization (1 µg siRNA, *n* = 3). Particle size (F) and PDI (G) of LNPs before and after lyophilization with sucrose or glucose as cryoprotectant (*n* = 3).

After obtaining the ICLs, each ICL was formulated into LNPs using a range of weight ratios. The FDA‐approved Onpattro LNP was similarly formulated with Dlin‐MC3‐DMA and characterized as described [22, 23]. In general, most synthesized lipids could form LNPs with sizes smaller than 200 nm and surface charge close to neutral (Table S1). The LNPs were then prepared with luciferase siRNA to evaluate the efficiency of gene silencing in vitro. Out of 50 lipids, 43 lipids showed varying degrees of gene silencing (Figure 4A,B). Several lipids displayed levels of gene silencing comparable to that of Dlin‐MC3‐DMA (∼80%). Overall, lipids with linker III showed higher knockdown efficiency in vitro, especially those with head A. Among the lipids that were comparable to Dlin‐MC3‐DMA in gene silencing, most did not show significant toxicity (cell viability ≥ 80%) as determined by BCA assay (Figure S5).

As an important factor that could affect the gene silencing ability of LNPs, the cellular uptake of selected LNPs was studied. HepG2 cells were treated with LNPs encapsulating Cy5.5‐labeled siRNA, and the cells were then harvested at 2 h after the treatment for flow cytometry. Four lipids were selected for this study due to their in vivo activity as described later in detail. It was shown that BIII2 LNP had the highest level of cellular uptake, followed by BIII6, as reflected by the mean fluorescence intensity within the Cy5.5‐positive cell population (Figure 4C). EIII2, EIII6, and Dlin LNPs showed comparable cellular uptake, but lower than that of BIII2 and BIII6. This could be attributed again to the protonation ability of lipids, and higher density of positive charge in head B featuring secondary amines in comparison to head E featuring piperazine and Dlin with tertiary amines. Particularly, as a known cationic lipid, DOTAP LNP also showed comparable uptake to BIII2 and BIII6. However, despite the differences in cellular uptake of all LNPs, the in vitro knockdown efficiency of these LNPs was quite comparable. These findings suggest that post‐uptake processes, particularly endosomal escape, determined by the ability of LNPs to disrupt biological membranes under acidic pH, may play a pivotal role in siRNA LNPs‐mediated gene silencing.

To examine the intracellular trafficking of LNPs, confocal microscopy was used. Cells were treated with LNPs encapsulating Cy5.5‐labeled siRNA, and images were taken at various time points. Endosomes/lysosomes were stained with LysoTracker Green. Figure 4D shows the images taken at 4 h after treatment with BIII2 and Dlin‐MC3‐DMA LNPs. Colocalization of Cy5.5 and LysoTracker Green was clearly visualized, suggesting uptake of LNPs via endocytosis. In addition, diffuse distribution of Cy5.5 in the cytosol was observed, suggesting escape of siRNA or siRNA LNPs from endosomes/lysosomes into cytoplasm following cellular uptake. Similar results were observed for BIII6, EIII2, EIII6, and DOTAP LNPs at different timepoints (Figures S6–S8).

All LNPs showed good stability during the lyophilization process in the presence of glucose or sucrose as a cryoprotectant. After reconstitution, the reconstituted LNPs retained gene‐silencing activity comparable to freshly prepared formulations, and no notable changes were detected in particle size or PDI (Figure 4E–G).

### 2.5 In Vivo Gene Silencing and Fusogenicity of LNPs

**FIGURE 5:**
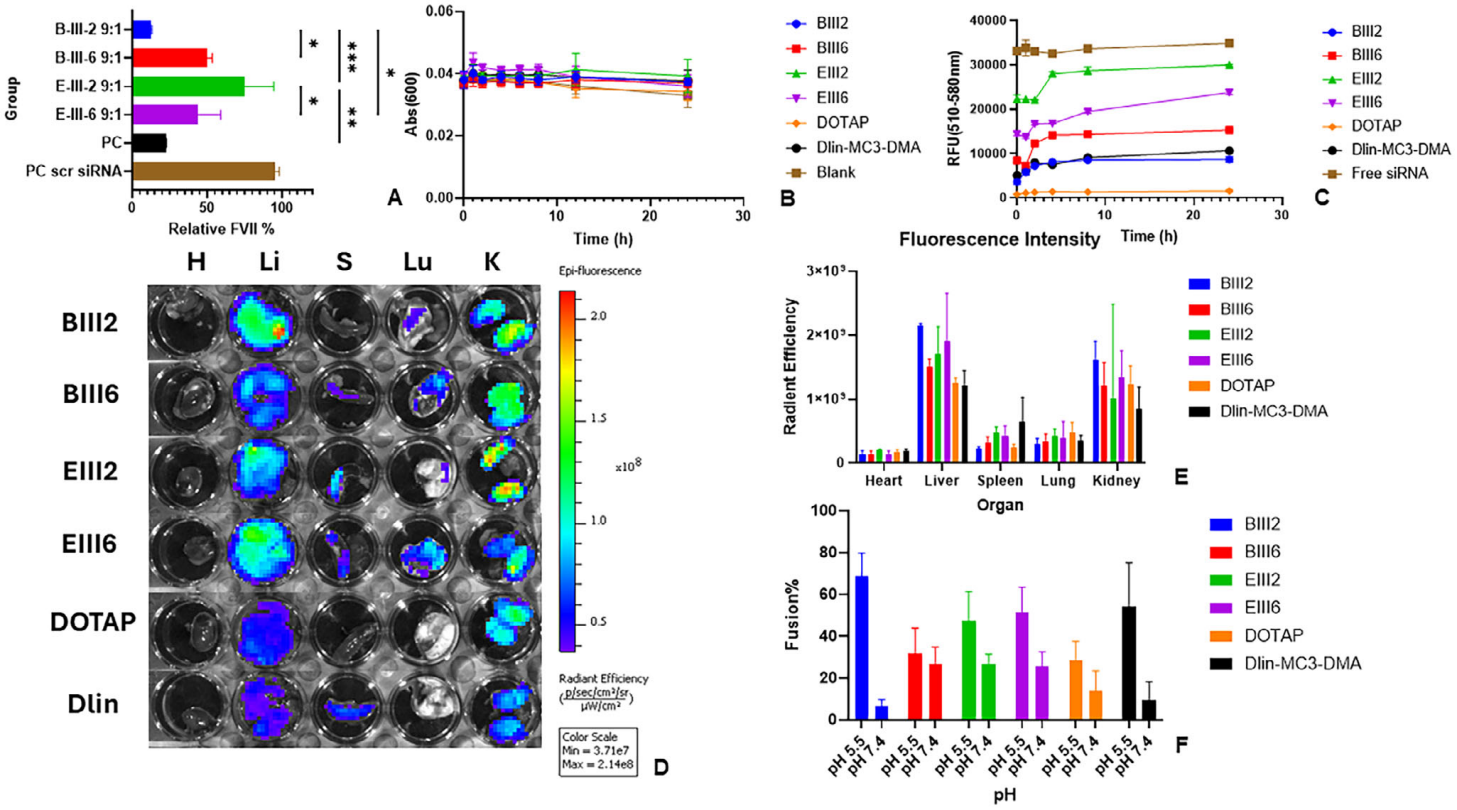
(A) FVII knockdown efficiency of selected LNPs in vivo (2 mg/kg FVII‐siRNA, *n* = 3). (B) UV–vis absorbance of mouse serum at 600 nm as an indicator for LNP‐induced serum protein aggregation monitored for 24 h (*n* = 3). (C) Fluorescence intensity of LNPs co‐incubated with 30% FBS was monitored for 24 h (*n* = 3). (D) Representative biodistribution images of LNPs. Dlin—Dlin‐MC3‐DMA lipid; H—Heart; Li—Liver; S—Spleen; Lu—Lung; K—Kidney. (E) Quantification of fluorescence intensity in major organs (*n* = 3). (F) Results of membrane fusion test using FRET assay (*n* = 3).

Based on in vitro screening results, lipids showing higher than 60% of gene knockdown were further tested in vivo for their ability to knock down FVII following an established method [24, 25]. FVII is produced by the liver, the organ that most reported LNPs intrinsically target. Surprisingly, out of 33 lipids, only four lipids showed a greater than 40% FVII gene silencing following a single dose. Notably, lipids showing FVII silencing all featured linker III, and none of the lipids synthesized with linker I was effective. Among the four effective lipids, lipid BIII2 was comparable to Dlin‐MC3‐DMA (the positive control, PC) in FVII silence (Figure 5A). Most reported linear ICLs that are effective in siRNA delivery have at least one double bond, or one degree of unsaturation, which is believed to enhance the fusion of LNPs with biological membranes to facilitate endosomal disruption and the subsequent release of the nucleic acid cargo into the cytoplasm [26, 27]. Our data were, in general, in line with these findings. For lipid BIII2, the addition of an extra double bond reduced the knockdown efficiency from 85% to 50%. On the other hand, for lipid EIII2, an extra double bond improved the knockdown efficiency when comparing EIII2 and EIII6. Typically, an effective ICL would feature a tertiary amine for binding with nucleic acid via electrostatic interactions, but BIII2, the most effective ICL in our series, featured secondary amines. Secondary amines are usually considered stronger than tertiary amines in terms of providing a positive charge, which may explain why BIII2 performed significantly better than EIII2 featuring tertiary amines in the piperazine ring. Clearly, while the structure of head, linker, or tail, each may affect the performance of lipids, the exact combination of the 3 motifs may be equally or more important in determining the activity of siRNA LNPs. Dose‐escalation toxicity study showed that LNPs were well‐tolerated at siRNA dosage of 8 mg/kg, equivalent to an ICL dosage of 400 mg/kg, as all groups showed less than 10% of body weight change (Figure S9).

One factor that could contribute to the gene silencing activity of LNPs in vivo is the colloidal stability of LNPs in biological fluids, especially in blood. The four identified lipids with distinct activity in vivo were further studied for their stability in serum. We first assessed the sensitivity of these LNPs to serum‐mediated aggregation by monitoring turbidity over 24 h after incubation with mouse serum, measured by UV–vis absorbance at 600 and 660 nm. No significant turbidity changes were observed during the entire monitoring period. Similar results were obtained when the LNPs were incubated with 10% FBS (Figure 5B; Figure S10).

The colloidal stability of LNPs following serum exposure was further tested using a Qubit assay, which employs target‐selective fluorescent dyes that emit only when bound to RNA. The binding between fluorescent dye and RNA relies on both electrostatic interactions and non‐covalent binding, like intercalation or groove binding, and strong fluorescence may only be produced when both interactions are in play simultaneously [28]. When the ICL binds to the RNA, the phosphate groups in RNA are no longer accessible to the fluorescent dye, leading to very weak fluorescence emitted. Increased fluorescence intensity indicates reduced siRNA packing within the LNPs after interacting with serum. All LNPs displayed an overall trend of increasing fluorescence, suggesting possible structural loosening or partial disruption—consistent with the generally loose and porous architecture of LNPs (Figure 5C). Notably, BIII2‐ and BIII6‐based LNPs showed much lower baseline fluorescence intensities than EIII2‐ and EIII6‐based LNPs. These differences may arise from the distinct amine headgroups: BIII2 and BIII6 contain TEPA with secondary amines, whereas EIII2 and EIII6 contain piperazine with tertiary amines. Because secondary amines typically exhibit stronger protonation than tertiary amines, they may bind siRNA more tightly through electrostatic interactions. This tighter binding would reduce dye accessibility to the encapsulated siRNA, leading to lower fluorescence signals throughout the experiment. Interestingly, the fluorescence profile of the best‐performing BIII2 LNPs closely resembled that of Dlin‐MC3‐DMA–based LNPs.

Other than colloidal stability, tissue accumulation is another critical early step for siRNA LNP–mediated gene silencing in vivo that needs to be examined as well. It is well established that the tissue tropism of LNPs is influenced by serum proteins adsorbed onto their surface—the so‐called protein corona [29, 30, 31]. Although the underlying mechanisms are not well understood, both the quantity and composition of protein coronas are affected by several factors, including the structure of the ICLs [32, 33, 34]. To better understand the mechanisms responsible for the different levels of in vivo gene knockdown achieved by our LNPs, we evaluated their tissue distribution using Cy5.5‐labeled siRNA and IVIS imaging (Figure 5D,E). Notably, BIII2, the most potent lipid, appeared to show the highest level of liver accumulation. EIII6 showed a comparable degree of hepatic accumulation but was significantly less effective in vivo, whereas Dlin‐MC3‐DMA lipid displayed relatively low liver accumulation despite its strong gene‐silencing efficiency. We acknowledge that ex vivo fluorescence imaging provides overall liver distribution but does not provide information on LNP uptake by distinct cell populations in the liver. Moreover, factors beyond tissue accumulation play critical roles in determining gene‐silencing efficacy.

After LNPs reach the target cells, their ability to successfully release siRNA cargo for gene silencing depends largely on endosomal escape or membrane disruption, which can be affected by their fusogenicity [35, 36]. The fusogenicity of LNPs was evaluated using a FRET assay based on the distance‐dependent energy transfer between two fluorescent probes [37]. In this assay, endosome‐mimicking nanovesicles (EM‐NVs) containing FRET probes were prepared. Disruption of the EM‐NVs by LNPs increases the distance between the donor fluorophore (NBD‐PE) and the acceptor fluorophore (Rho‐PE), resulting in decreased rhodamine fluorescence.

Except for BIII6 LNPs, all tested LNPs exhibited enhanced membrane fusion at pH 5.5, mimicking the acidic endosomal/lysosomal environment. Among them, BIII2 showed the highest fusion percentage under acidic conditions. Notably, BIII2 also displayed the largest difference in fusogenicity between pH 7.4 and 5.5, suggesting accelerated release of siRNA or siRNA‐loaded LNPs from endosomes/lysosomes into the cytosol following endocytosis (Figure 5F). Interestingly, the fusogenic profile of BIII2 LNPs—both the absolute fusogenic activity at pH 5.5 and the pH‐dependent increase—was comparable to that of DLin‐MC3‐DMA LNPs, which may explain its favorable safety profile and robust in vivo gene silencing efficacy.

**FIGURE 6:**
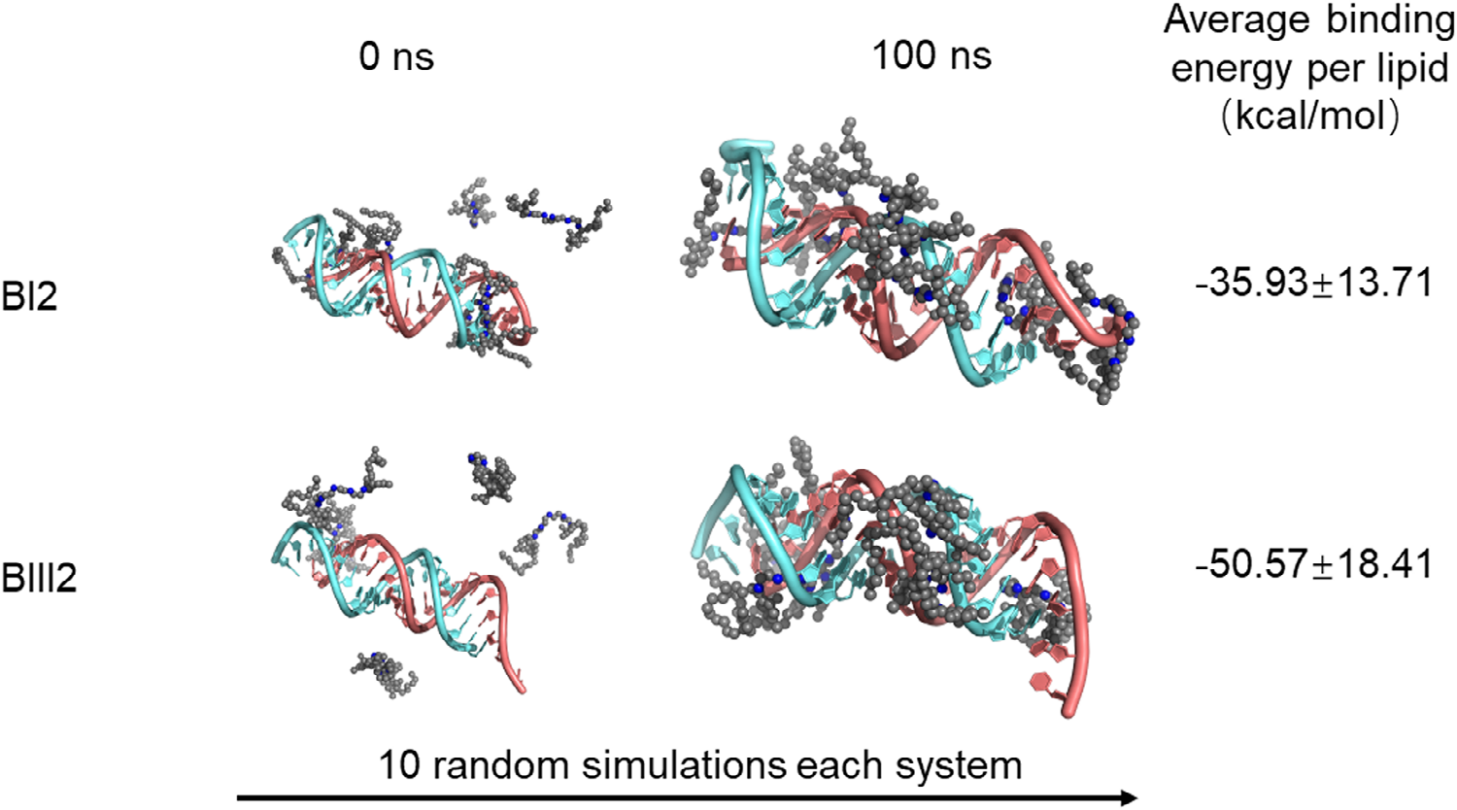
Binding free energy of siRNA and ionizable cationic lipids BI2 and BIII2, calculated using MM/PBSA Using MM/PBSA method, and the diagram illustrating siRNA/lipid complexation.

We also applied a computational method to investigate how the structure of lipids may affect the in vivo performance of the lipids in our library (Figure 6). Herein, we mainly focused on the effect of the linker due to its critical role in gene silencing, as shown in our in vivo study: the change of linker III in BIII2 to linker I in BI2 completely abolished its gene silencing efficiency. We constructed two distinct siRNA‐loaded lipid systems, BI2 and BIII2, based on their experimental ICL–to–siRNA ratios. Notably, both systems contain three secondary amines. Each system was equilibrated using the molecular dynamics (MD) engines in the AMBER software [38], followed by ten (10) independent MD simulations initiated with different random seeds. The resulting trajectories were analyzed using the MM/PBSA method to calculate the binding affinities [39, 40]. BIII2 exhibited significantly stronger binding to siRNA (−50.57 ± 18.41 kcal/mol) than BI2 (−35.93 ± 13.71 kcal/mol). Compared to BI2, the additional hydroxyl group in BIII2 likely plays a significant role in enhancing its interaction with siRNA, as it can serve as an extra hydrogen bond donor. On the other hand, it is apparent that the lack of a ketone carbonyl group in BIII2 has less impact when interacting with siRNA. A more stable complex structure, as observed with BIII2, is crucial for efficient siRNA delivery toward target cells. To evaluate the ionization state of the linker amines under physiological conditions, we employed the EPIK (Empirical pKa and Ionization Calculator) module within the Schrödinger suite to predict their pKa [41]. The EPIK methodology utilizes a quantitative structure‐property relationship (QSPR) model to provide rapid and reliable estimations of molecular ionization in solution.

Our calculations showed that linkers B, E, and D amines each contain at least two nitrogen atoms with pKa values exceeding 8.0, indicating that these amines will be predominantly protonated and thus effectively carry a positive charge at the physiological pH of 7.4. In contrast, A and C linkers with lower pKa values are less likely to maintain a stable positive charge, leading to instability in blood plasma (Table 1).

**TABLE 1:** Calculation of pKa of nitrogen atoms in LNPs.

Molecules	Structures	pKa
**AIII**	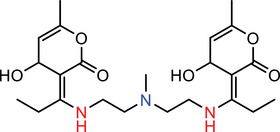	**N** (Red) = ‐2.40 ± 0.59 **N** (Blue) = 7.16 ± 1.04
**BIII**	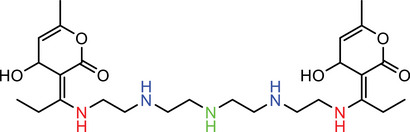	**N** (Red) = ‐2.34 ± 0.59 **N** (Blue) = 8.74 ± 0.56 **N** (Green) = 8.93 ± 0.56
**CIII**	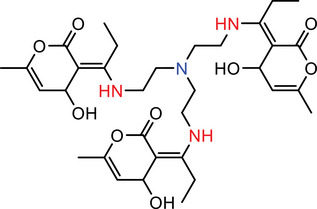	**N** (Red) = ‐2.49 ± 0.59 **N** (Blue) = 5.99 ± 1.47
**DIII**	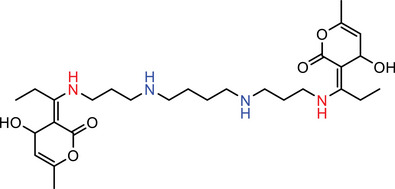	**N** (Red) = ‐2.39 ± 0.59 **N** (Blue) = 10.54 ± 1.47
**EIII**	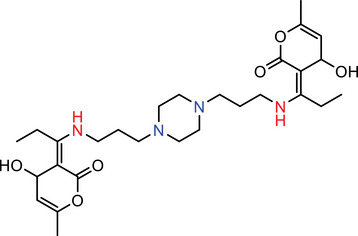	**N** (Red) = ‐2.72 ± 0.59 **N** (Blue) = 7.72 ± 2.22

A more detailed comparison among the high‐performing linkers highlights a key structural advantage of linker B. While linker D exhibits a higher individual pKa value, linker B features three potential charge centers, whereas linkers D and E only have two. At pH ≤ 7.4, linker B is predicted to form stronger interactions with nucleic acids, due to the greater number of potential hydrogen bonds and more extensive electrostatic interactions afforded by its multiple protonated sites. While it's well‐known that the apparent pKa of effective ICLs typically lies between 6.5 to 7, our modeling could provide more insights as it calculated the pKa of individual nitrogen atoms rather than that of the entire ICL molecule. Such a method may more accurately reflect the protonation status and binding affinity with nucleic acids of ICLs. To summarize, ICLs containing lower‐pKa amines may promote stronger nucleic acid association and increased LNP stability, thereby enhancing in vivo transfection efficiency. We acknowledge that, while our modeling approach may help inform the design and identification of ICLs for in vivo siRNA delivery, its applicability requires additional experimental validation. Nevertheless, our experimental data showing pH‐dependent changes in the fusogenic activity of LNPs toward endosome‐mimicking vesicles (Figure 5F) support the biological relevance of the pKa values of our ICs in the context of LNP‐mediated siRNA delivery. In addition, we performed preliminary molecular dynamics simulations demonstrating LNP fusion with a lipid bilayer (Figure S12 and Supporting Video). Further simulations examining ICL packing and inverted micelle formation will be the subject of future studies.

In conclusion, this study has demonstrated the feasibility of applying β‐triketone‐based click chemistry in synthesis of ICLs in large quantities and in a short time. The availability of a library of ICLs of new structural features may further enrich our understanding of their SAR. It may also lead to the development of improved LNPs for more effective delivery of nucleic acids including siRNA.

## 3 Materials and Methods

### 3.1 Chemicals and Reagents

Dlin‐MC3‐DMA lipid, 1,2‐distearoyl‐sn‐glycero‐3‐phosphocholine (DSPC), and 1,2‐dimyristoyl‐rac‐glycero‐3‐methoxypolyethylene glycol‐2000 (DMG‐PEG2000) were purchased from Avanti Polar Lipids (AL, USA). Ethanol and RNase‐free water were purchased from Fisher Scientific (NH, USA). All other chemicals used in this study were purchased from Sigma–Aldrich (MO, USA) unless specified otherwise. All kits used, such as Pierce firefly luciferase glow assay kit, Pierce BCA protein assay kit, and FVII assay kit, were purchased from Thermo Fisher Scientific (MA, USA) unless specified otherwise.

### 3.2 Cell Line and Cell Culture

Human hepatoma cell line, HepG2, was kindly provided by Dr. Wen Xie in the School of Pharmacy, University of Pittsburgh. A luciferase‐expressing HepG2 cell line (HepG2 Luc+) was established by transfecting normal HepG2 cells with mRNA encoding EGFP‐luciferase using lipofectamine 3000 as per the manufacturer's instructions. The EGFP‐luciferase positive cells were selected and separated by flow cytometry.

### 3.3 General Synthesis of Ionizable Cationic Lipids (ICLs)

The synthesis of ionizable cationic lipids was completed in two steps [10, 12]. The first step is to conjugate the linker and tail with the help of a condensing agent to form tri‐ketone compounds. The second step is to mix di/tri‐amine with tri‐ketone lipid to form an imine structure without purification.

For the first step, 10.5 mmol of β‐triketone linker, 10 mmol of tail,12 mmol of N,N'‐Dicyclohexylcarbodiimide (DCC), and 15 mmol of 4‐Dimethylaminopyridine (DMAP) were dissolved in 40 mL dichloromethane (DCM), and allowed to react under stirring at room‐temperature for 24 h. The product was filtered, then washed with 1 m HCl thrice and brine once before purification by silica gel chromatography (hexane: ethyl acetate = 8:1).

For the second step, 1 mmol di‐amine (Head A, B, D, and E) was dissolved in 10 mL DCM and mixed with 2.1 mmol of β, β’‐triketone lipid obtained in the first step. Alternatively, 1 mmol of tri‐amine (Head C) was dissolved in 10 mL DCM and mixed with 3.1 mmol of β, β’‐triketone obtained in the first step. The mixture was stirred at room‐temperature for 15 min, and the solvent was removed using a rotatory evaporator to obtain the final product of ionizable cationic lipid. The crude final product was used for the preparation of LNPs without purification [42, 43, 44].

### 3.4 Demonstration of Click Chemistry for Synthesis of β, β’‐Triketone‐Lipid and Its Degradation

Glycine, methyl glycine, dimethyl glycine, and 2‐acetyl dimedone were prepared as 10 mg/mL solutions in H_2_O. Glycine and its derivatives were each mixed with 2‐acetyl dimedone at a molar ratio of 1:1, and the reaction mixture was immediately dipped into liquid nitrogen and transferred to ‐80°C freezer prior to lyophilization. The samples were reconstituted in D_2_O to obtain ^1^H‐NMR spectrum.

For the degradation study, glycine and 2‐acetyl dimedone stock solutions were prepared in H_2_O. Glycine and 2‐acetyl dimedone were mixed at a molar ratio of 1:1 in a centrifuge tube and allowed to react for 5 min. Then the tube was instantly dipped into liquid nitrogen at each timepoint and transferred to ‐80°C freezer. The samples were lyophilized overnight prior to resuspension in CDCl_3_, followed by centrifugation. The supernatant was taken for a ^1^H‐NMR spectrum. The detection of 2‐acetyl dimedone was defined as the appearance of its proton peaks with the correct integrated ratio.

### 3.5 Preparation and Characterization of LNPs

The LNPs loading siRNA was prepared as described in previous publications [45, 46, 47]. In short, an ethanol solution of lipids was prepared at a ratio of 50:38.5:10:1.5 (Ionizable cationic lipid: cholesterol: DSPC: DMG‐PEG2000) at an ICL concentration of 10 mg/mL, and siRNA was dissolved in 10 mm citric acid. The synthesized ICLs and siRNA were mixed at an ICL/siRNA ratio of 25:1 (w/w) using a microfluidic machine (Precision Nanosystems, Canada). Dlin‐MC3‐DMA LNP was prepared at an ICL/siRNA ratio of 10:1 (w/w) using the same method [24]. The resulting LNPs were washed using ultra‐centrifugation thrice at 12 500 G for 10 min and diluted to an appropriate concentration for measurement of size and zeta potential using Zetasizer Nano‐ZS (Malvern Panalytical, USA). For lyophilization stability evaluation of LNPs, cryoprotectant, including sucrose and glucose solution, was added to yield LNPs, at a final concentration of 10% (w/v). The LNPs were then frozen, lyophilized, and re‐suspended in water before further testing.

### 3.6 Evaluation of Gene Knockdown Efficiency by siRNA‐Loaded LNPs

LNPs fabricated using synthesized ionizable cationic lipids were prepared as described in the previous section. HepG2 Luc+ cells were seeded in 96‐well plates in a density of 4000 cells/well, and each well of cells was treated with LNPs equivalent to 0.25 µg luciferase siRNA for 48 h. Dlin‐MC3‐DMA LNPs were prepared as previously reported in the literature. HepG2 Luc+ cells treated with Dlin‐MC3‐DMA LNPs loading luciferase siRNA served as a positive control, while those treated with an equal amount of RNase‐free water served as a negative control. The expression of luciferase in cells was determined using the Pierce firefly luciferase glow assay kit as per the manufacturer's instructions. In short, cells were washed with PBS thrice to remove detached dead cells, and then were lysed with lysis buffer for 15 min. The cell lysate was centrifuged at 13,300 G for 10 min, and 20 µL of supernatant was transferred to an opaque 96‐well plate and added with 50 µL luciferin working solution. The plate was incubated at RT for 5 min prior to measurement of luminescence using a plate reader (SYNERGY H1, BioTek, USA).

### 3.7 Protein Quantification Assay

A protein quantification assay was performed to normalize luminescence readings and reflect cell viability. Pierce BCA protein assay kit (Thermo Fisher Scientific, USA) according to the manufacturer's instructions, and the UV absorbance was measured using a plate reader (SYNERGY H1, BioTek, USA) at 562 nm. Briefly, cells were washed with PBS thrice to remove detached dead cells, and then were lysed with lysis buffer for 15 min. The cell lysate was centrifuged at 13 300 G for 10 min, and 20 µL of supernatant was transferred to a 96‐well plate and added with 180 µL BCA assay buffer. A standard curve from 12.5 µg/mL to 200 µg/mL was established by using the manufacturer's standard solutions ranging from 125 to 2000 µg/mL using the same method.

### 3.8 Evaluation of Gene Knockdown Efficiency by siRNA‐Loaded LNPs In Vivo

The animal protocol followed in this study was approved by IACUC at the University of Pittsburgh. All LNPs used for tests to evaluate gene knockdown efficiency in vivo were prepared as described in previous sections, and plasma FVII level in mice was determined using an established method [24, 25]. In short, C57BL/6 mice were treated with siRNA LNPs via I.V. injection at a siRNA dosage of 2 mg/kg. The mice were sacrificed 24 h after the treatment, and the blood was collected, and the plasma was isolated under centrifugation. The plasma FVII level was measured using BIOPHEN FVII assay kit (HYPHEN BioMed, France) as per the manufacturer's instructions. The mice in the non‐treatment group served as a negative control to establish 100% baseline for plasma FVII level.

### 3.9 Serum Stability of LNPs

The stability of LNPs in serum was tested as described previously [48, 49]. The aggregation state of LNPs were determined by measuring UV–vis absorbance of LNP mouse serum or 10% FBS co‐incubated with LNPs. In short, 100 µL of mouse serum or 10% FBS was added to each well of a clear 96‐well plate, followed by the addition of LNPs containing 100 ng of siRNA. Absorbance was measured at 600 and 660 nm at different timepoints using a plate reader (SYNERGY H1, BioTek, USA).

### 3.10 Qubit Assay

To determine the stability of LNPs and their protection of encapsulated siRNA from biological fluid, the Qubit assay was performed using Qubit ssDNA Assay Kit (Invitrogen, USA) according to the manufacturer's instructions. Briefly, a working solution was first prepared by diluting the Qubit ssDNA Reagent 1:200 in Qubit ssDNA Buffer. After that, 10 µL of LNPs prepared as described in the previous section were added to 190 µL of working solution. The fluorescence was then measured at different timepoint using a Qubit 4.0 fluorometer (Invitrogen, USA).

### 3.11 Lipid Fusion Analysis by FRET Assay

The FRET assay was used to determine the fusogenicity of LNP. FRET‐conjugated fluorescent probes NBD‐PE (N‐(7‐nitrobenz‐2‐oxa‐1,3‐diazol‐4‐yl)‐1,2‐dihexadecanoyl‐sn‐glycero‐3‐phosphoethanolamine, triethylammonium salt) and Rho‐PE (Lissamine rhodamine B 1,2‐dihexadecanoyl‐sn‐glycero‐3‐phosphoethanolamine, triethylammonium salt) were purchased from Thermo Fisher Scientific. Endosome‐mimicking nanovesicles (EM‐NV) were prepared by mixing DOPS:DOPC:DOPE:NBD‐PE:Rho‐PE in a molar ratio of 25:25:48:1:1 in chloroform. The mixture solution was dried to yield a thin‐film, and re‐hydrated in PBS under sonication for 20 min. The concentration of total lipid was kept at 1 mm. Following the addition of PBS at pH 5.5 or 7.4 to opaque 96‐well plates (100 µL per well), 2 µL of EM‐NV and 2 µL of LNPs were added to each well. After incubation for 5 min at 37°C, the fluorescence (F) of RhO‐PE was measured using a plate reader (Ex: 470 nm; Em: 590 nm) (SYNERGY H1, BioTek, USA). Negative control (F_0_) consisted of only EM‐NV in PBS at pH 5.5 or 7.4. Positive control (F_min_) consisted of EM‐NV incubated with Triton X‐100 in PBS at pH 5.5 or 7.4. The percentage lipid fusion was then calculated as $Lipid\;fusion\%=\frac{F_{0}-F}{F-F_{min}}\;\times 100\%$.

### 3.12 Biodistribution of LNPs

For biodistribution profiles of LNPs, the LNPs were prepared as described in previous sections. In short, mice were treated with LNPs containing cy5.5‐labeled (0.1 mg/kg siRNA). The mice were then euthanized 24 h post treatment, and major organs were collected and imaged ex vivo using In Vivo Imaging System (IVIS) (Lumina XR, Caliper LifeSciences, USA). The fluorescence intensity was quantified using Living Image software.

### 3.13 Cellular Uptake of LNPs

The cellular uptake of LNPs was examined using flowcytometry. In short, HepG2 cells were seeded into a six‐well plate (400 000 cells per well). When reaching 80%–90% confluency, cells were treated with LNPs containing cy5.5‐labeled siRNA (2 µg siRNA per well), then harvested 2 h after treatment to obtain a single cell suspension. The cells were analyzed using an LSR Fortessa laser scanning cytometer (BD Sciences, USA) to identify populations with LNP uptake. The data were then processed using Flow Jo 10.0 software for quantitative determination of siRNA uptake in cells.

### 3.14 Confocal Laser Scanning Microscopy for Intracellular LNP Trafficking

Confocal laser scanning microscopy (CLSM) was used to track the intracellular trafficking of LNPs in vitro. HepG2 cells were seeded in chamber slides (4000 cells per well), and treated with LNPs containing cy5.5‐labeled siRNA (0.5 µg siRNA per well) 24 h later. At 1, 4, and 24 h post treatment, LysoTracker Green was added to each well according to the manufacturer's instructions to stain endosome. The cells were then fixed using 4% paraformaldehyde. Prior to CLSM imaging, the slide was covered with cover glass using DAPI‐containing mounting medium.

### 3.15 Reaction Barrier Calculation

Reactant and product geometries were fully optimized at ωB97X‐D/6‐31G^*^ with the SMD water model and tight SCF convergence (scrf = SMD opt freq SCF = tight). Harmonic‐frequency analyses at the same level confirmed the absence of imaginary modes and provided thermal corrections. Single‐point energies were refined at ωB97X‐D/def2‐TZVP. The Gibbs free energy of each species, G, was obtained as the sum of single‐point energy and thermal correction to Gibbs Free Energy: $G=E_{elec}^{def2TZVP}+\Delta G_{therm}^{6-31G^{*}}\left(298.15\;K\right)$.

### 3.16 *pKa* Analysis of Linkers in LNPs

Acid dissociation constants were derived from the thermodynamic cycle in Figure S11A by [50]:

$$\begin{array}{cc} pKa & =\left[G_{gas}\left[A^{-}\right]-G_{gas}\left[HA\right]+\Delta G_{solv}\left[A^{-}\right]\right.\\ & \;-\left.\Delta G_{solv}\left[HA\right]-270.9\right]\;\times 4186/RTln10 \end{array}$$

Initial structures were first minimized by MM2 in Chem3D. Gas‐phase geometries and enthalpies were obtained at the CBS‐QB3 level [51]. Solvated geometries and frequencies were re‐optimized at ωB97X‐D/def2‐TZVP/SMD. Solvation free energies, Δ*G_solv_*, were subsequently evaluated at M06‐2X/6‐31G^*^, a combination shown to yield highly reliable SMD hydration energies at moderate cost [52, 53]. The proton hydration free energy (270.9 kcal mol^−^^1^) was taken from experiment [54].

Linker I is derived from dimedone, which has an experimentally determined pKa value of 5.27. Due to the failure of direct calculation of pKa of amine on Linker I, we introduced Linker II, which originated from Meldrum's acid, whose experimental pKa value is 4.97 (Figure S11B) [55]. Our calculated results are in close agreement with these values, indicating that the computational method is reasonably reliable. We attempted to calculate the pKa of the nitrogen proton in the Schiff base substructures using molecular fragments. Direct pKa calculation for Linker I failed, because proton transfer occurred from the secondary amine to the oxygen in gas phase calculation. For Linker II, the calculated pKa fell within the range of 5–7, as anticipated. The pKa of the *cis* form was calculated to be 11.1. The calculation for the *trans‐* form failed due to the instability of the intermediate structure, like Linker I.

To further investigate relative value, we methylated the secondary amines to form quaternary ammonium groups and recalculated the pKa values (Figure S11C). Both Me‐II and Me‐III exhibited increased pKa values, due to weakened hydrogen bonding and electrostatic interactions, making it more difficult to stabilize the negative charge. Me‐I showed a slightly higher pKa than Me‐II, which is consistent with the experimental pKa values of dimedone and Meldrum's acid. The pKa of Me‐*cis*‐III is higher. This is related to its stability to pH during the delivery process of siRNA.

### 3.17 MM/PBSA Binding Free‐Energy Calculations

A siRNA duplex was modeled with AlphaFold 3 [56] and embedded in explicit TIP3P water together with six cationic lipid molecules, giving a negative: positive charge ratio of ≈ 3:1 in accordance with the formulation protocol. The OL3 force field was used for nucleic acids. Parameters for the lipids were generated with Antechamber/GAFF2: geometries were optimized at ωB97X‐D/6‐31G^*^ (SCF = tight), and RESP atomic charges were fitted to electrostatic potentials computed at HF/6‐31G^*^ with the Merz–Kollman scheme (Pop = MK iop(6/33 = 2,6/42 = 6)).

After steepest‐descent minimization, the system was heated to 298.15 K and equilibrated for 1 ns in the NPT ensemble. Ten independent production runs (100 ns each, 0.5 fs time step) were initiated from different velocity seeds. Binding free energies were evaluated with the MM/PBSA module of Amber 24, analyzing the final 25 ns of each trajectory and extracting one frame every 0.5 ns.

Interactions with BI2, BII2, and BIII2 were considered significant when the MM/PBSA binding energy was more negative than −15 kcal/mol, assuming each charged lipid carries three positive charges. For BII2 at higher pH, where each lipid is assumed to carry two positive charges due to reduced protonation based on pKa, a more relaxed threshold of −10 kcal/mol was used to define significant interactions. The average binding energy per lipid of BII2 is ‐44.76 ± 16.16 and ‐25.89 ± 8.38 kcal/mol at two different states, proving that the higher the charge number is, the stronger the combination will be.

### 3.18 Statistics and Data Analysis

For characterization of LNPs, each formulation was characterized with three different batches of LNPs. For the evaluation of gene knockdown efficiency, an experiment for each formulation was repeated three times. Protein quantification was completed using absorbance values from samples collected from treatment wells and calculated using the equation determined from the standard curve on the same plate established using the standards in the assay kit. All statistics were calculated using GraphPad Prism 10 using one‐way analyses of variance (ANOVA) followed by post‐hoc Dunnett's test. Statistical significance was set at *p* < 0.05, and data are reported as the mean ± SEM.

## Conflicts of Interest

The authors declare no conflict of interest.

## Supporting information




**Supporting File**: advs74560‐sup‐0001‐SuppMat.docx.

**Supporting File**: advs74560‐sup‐0002‐SuppVideo.mp4.

## Data Availability

The data that support the findings of this study are available from the corresponding author upon reasonable request.
