# Supplementary material for: β‐Triketone‐Based Ionizable Cationic Lipids Synthesized via Click Chemistry for siRNA Delivery
Source: Adv Sci (Weinh). 2026 Mar 30;13(26):e15482. doi: 10.1002/advs.202515482 (PMC13159103; doi:10.1002/advs.202515482)
Supplement: Supplementary file 1 — Supporting File: advs74560‐sup‐0001‐SuppMat.docx. [file ADVS-13-e15482-s002.docx]

**β-triketone-based Ionizable Cationic Lipids Synthesized via Click Chemistry for siRNA Delivery**

Huatian Li^1^, Haocheng Tang^2^, Yiqing Mu^1^, Shangyu Chen^1^, Paul Edward Floreancig^3^, Junmei Wang^2^*, Yixian Huang^1^*; and Song Li^1^*

1. Center for Pharmacogenetics, Department of Pharmaceutical Sciences, School of Pharmacy, University of Pittsburgh, Pittsburgh, PA 15261, USA
2. Computational Chemical Genomics Screening Center, Department of Pharmaceutical Sciences, School of Pharmacy, University of Pittsburgh, Pittsburgh, PA 15261, USA
3. Department of Chemistry, Dietrich School of Arts and Sciences, University of Pittsburgh, PA 15260, USA

*Corresponding authors

**Supporting Information**

**Table of Contents**

| **^1^H NMR Spectrums** | **3-7** |
| --- | --- |
| **Supplementary Figure 1** | **8** |
| **Supplementary Figure 2** | **9** |
| **Supplementary Figure 3** | **10** |
| **Supplementary Figure 4** | **11** |
| **Supplementary Figure 5** | **12** |
| **Supplementary Figure 6** | **13** |
| **Supplementary Figure 7** | **14** |
| **Supplementary Figure 8** | **15** |
| **Supplementary Figure 9** | **16** |
| **Supplementary Figure 10** | **17** |
| **Supplementary Figure 11** | **18** |
| **Supplementary Figure 12** | **19** |
| **Supplementary Table 1** | **20** |

**^1^H NMR Spectrums**


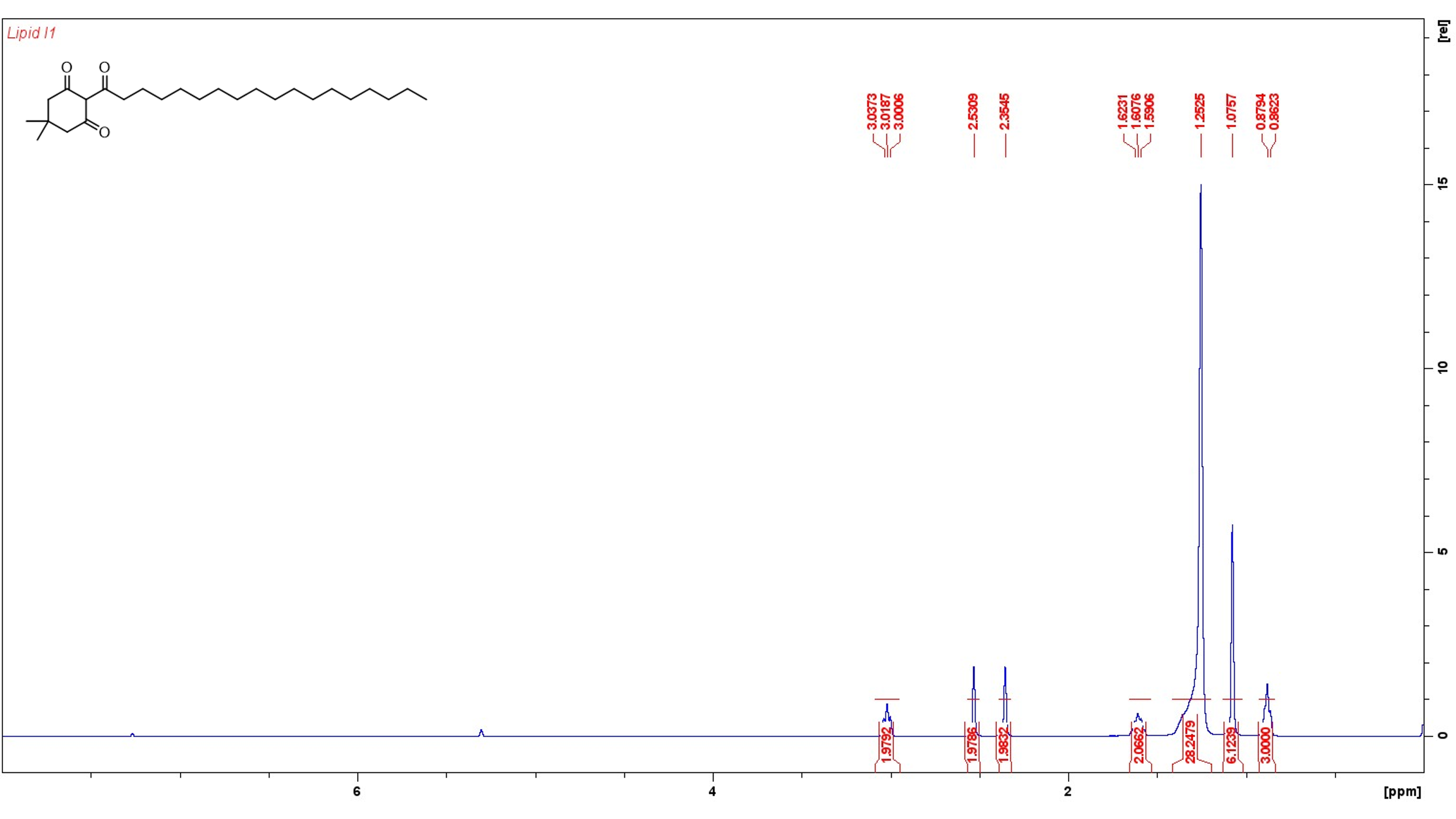


β, β’-triketone lipid I1 – Characterization Data: ^1^H NMR (400 MHz, CDCl_3_): δ 3.02 (t, 2H), 2.53 (s, 2H), 2.35 (s, 2H), 1.61 (t, 2H), 1.25 (m, 29H), 1.08 (s, 6H), 0.88 (t, 3H) ppm


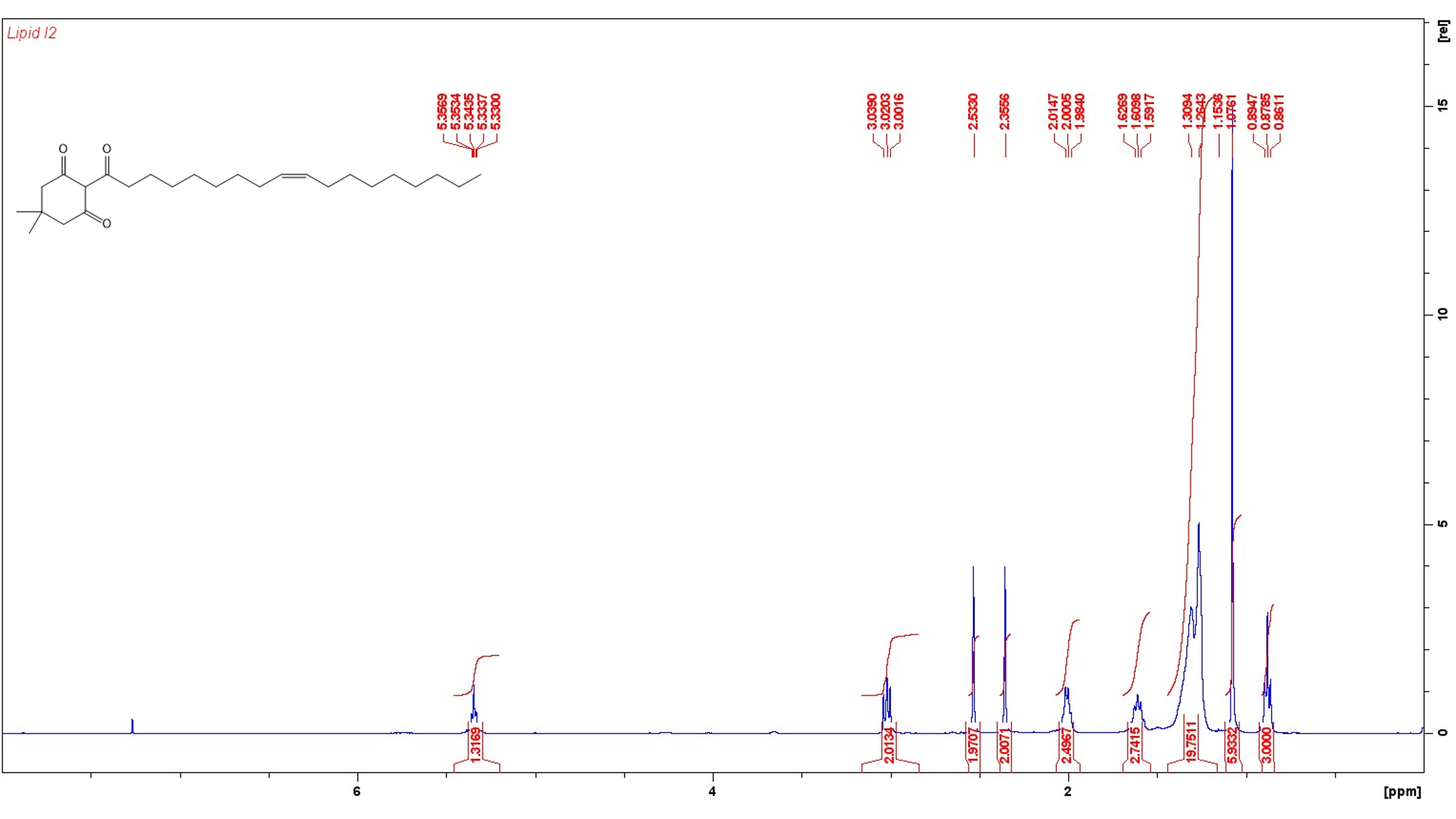


β, β’-triketone lipid I2 – Characterization Data: ^1^H NMR (400 MHz, CDCl_3_): δ 5.34 (m, 2H), 3.02 (t, 2H), 2.53 (s, 2H), 2.36 (s, 2H), 2.00 (t, 3H), 1.61 (t, 2H), 1.26 (m, 21H), 1.08 (s, 6H), 0.88 (t, 3H) ppm


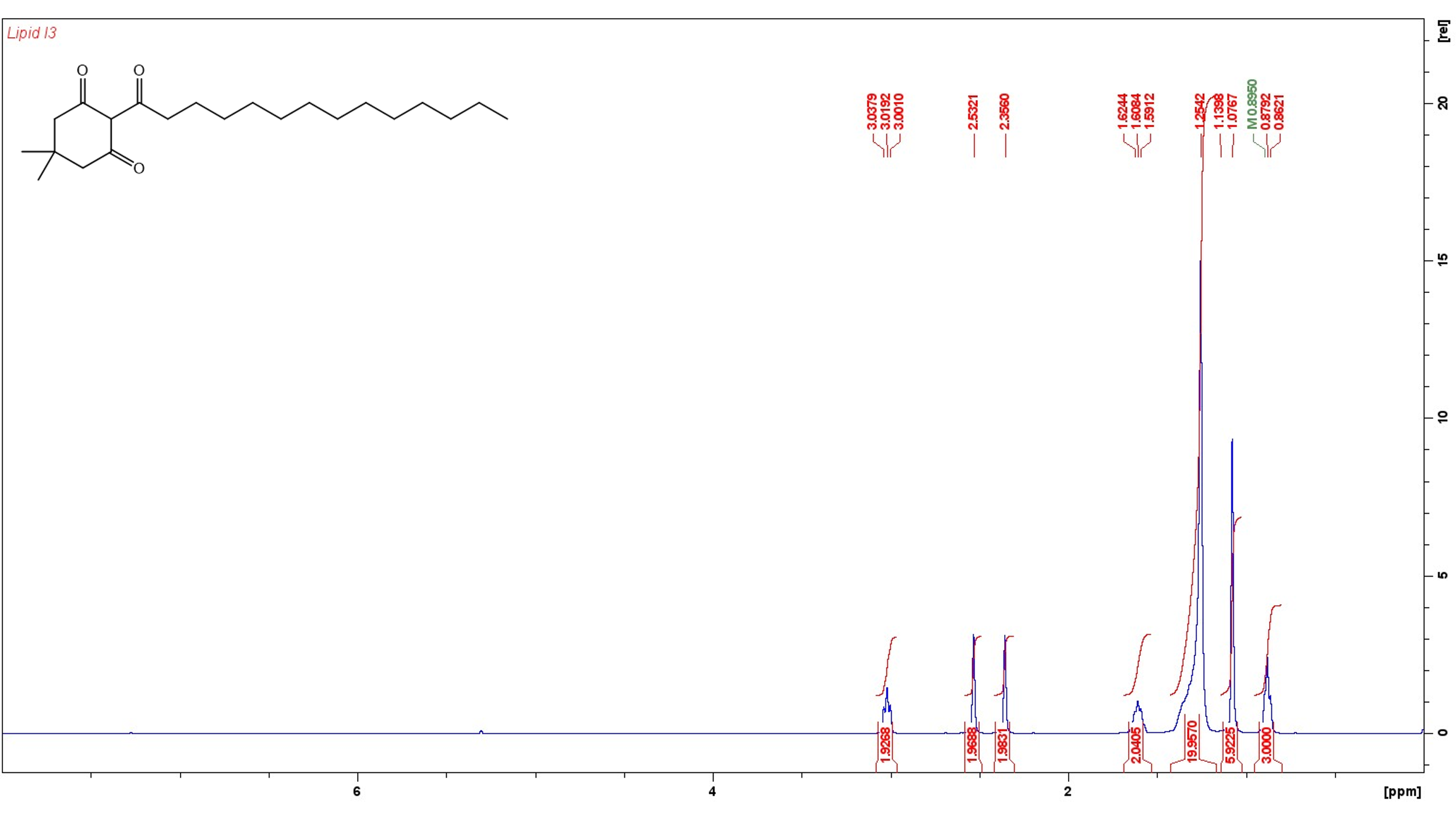


β, β’-triketone lipid I3 – Characterization Data: ^1^H NMR (400 MHz, CDCl_3_): δ 3.02 (t, 2H), 2.53 (s, 2H), 1.61 (t, 2H), 1.26 (m, 21H), 1.08 (s, 6H), 0.88 (t, 3H) ppm


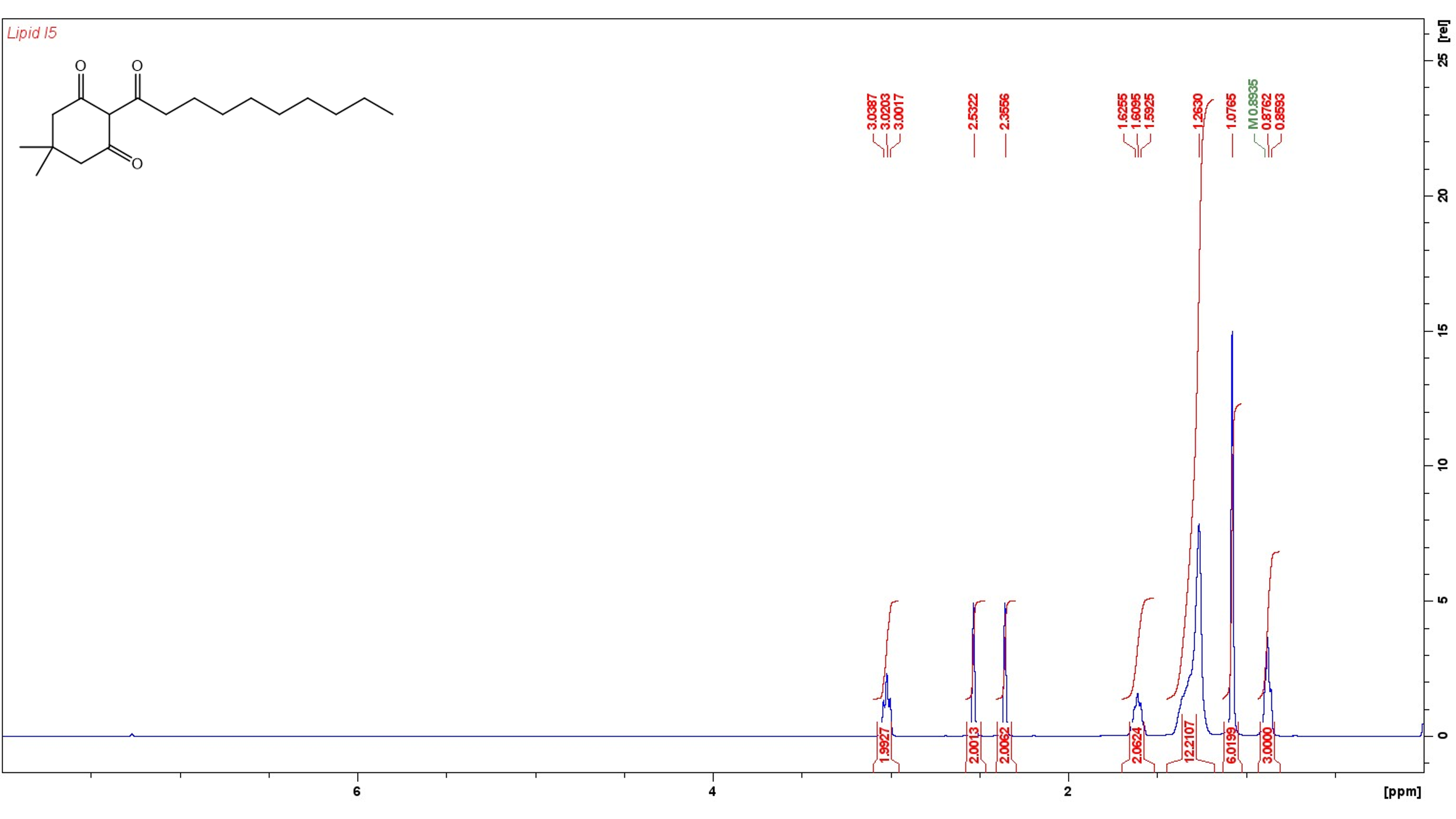


β, β’-triketone lipid I5 – Characterization Data: ^1^H NMR (400 MHz, CDCl_3_): δ 3.02 (t, 2H), 2.53 (s, 2H), 1.61 (t, 2H), 1.26 (m, 15H), 1.08 (s, 6H), 0.88 (t, 3H) ppm


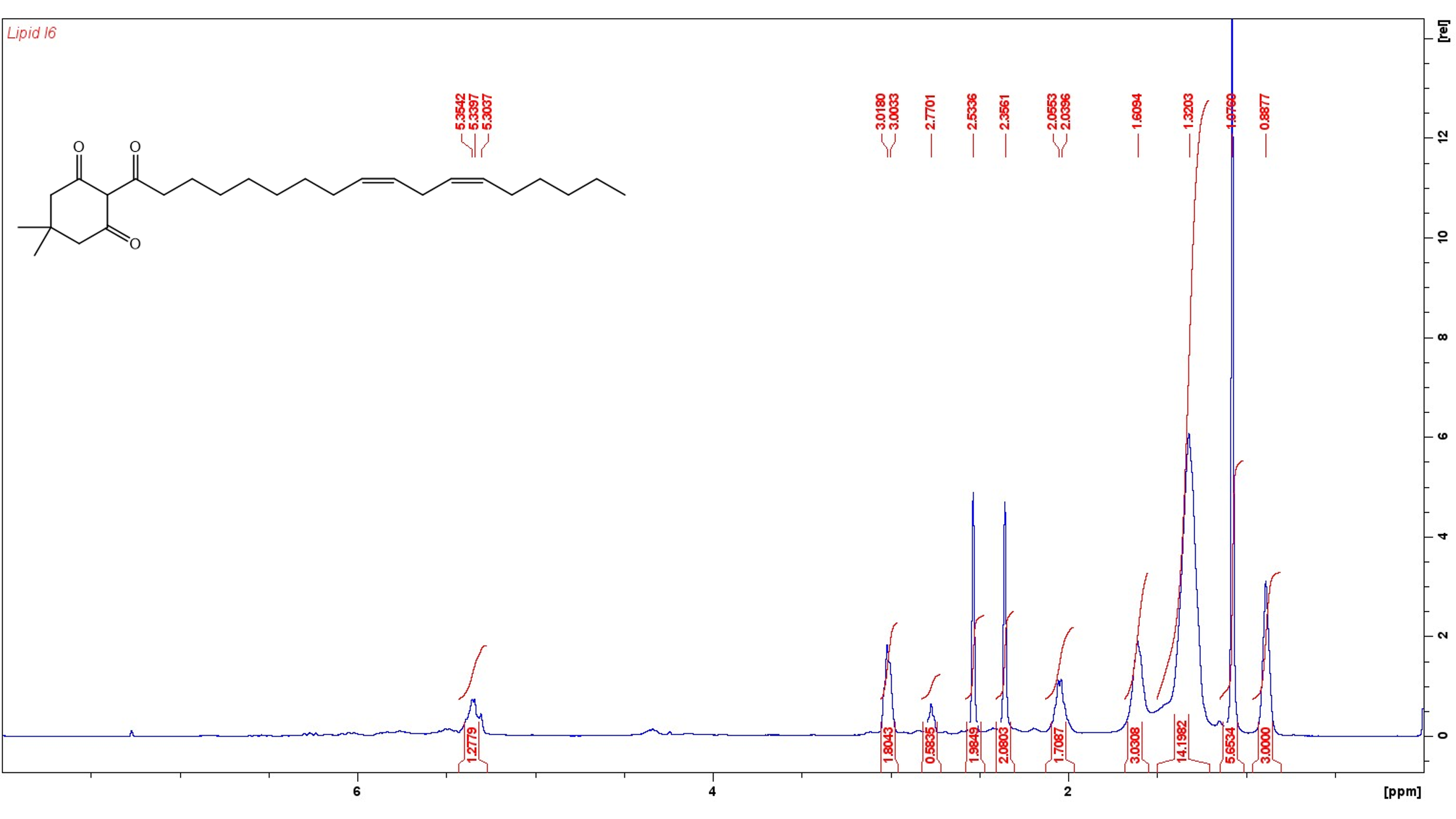


β, β’-triketone lipid I6 – Characterization Data: ^1^H NMR (400 MHz, CDCl_3_): δ 5.34 (m, 4H), 3.02 (t, 2H), 2.77 (m, 4H), 2.53 (s, 2H), 2.36 (s, 2H), 2.00 (t, 3H), 1.61 (t, 2H), 1.26 (m, 15H), 1.08 (s, 6H), 0.88 (t, 3H) ppm


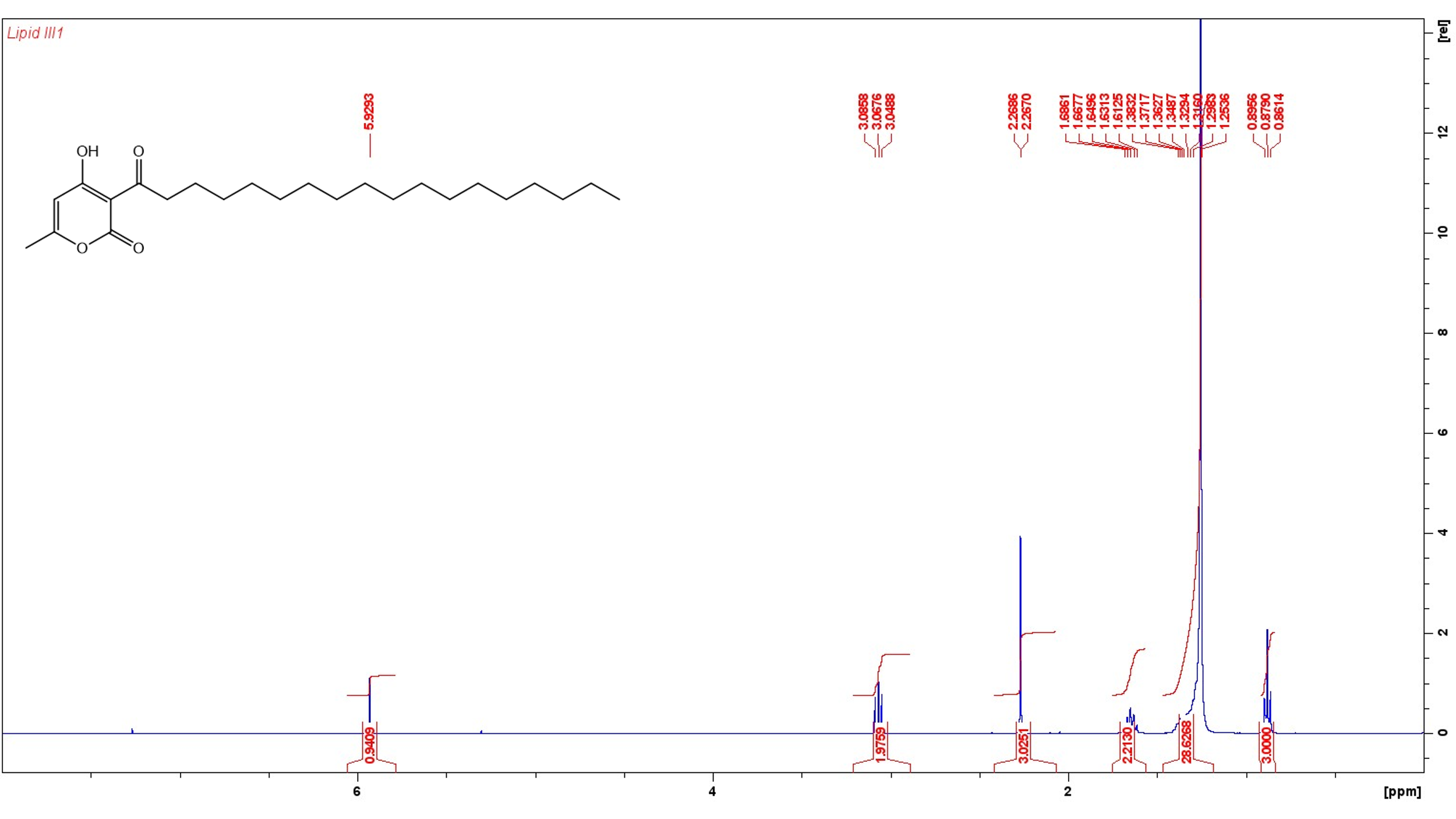


β, β’-triketone lipid III1 – Characterization Data: ^1^H NMR (400 MHz, CDCl_3_): δ 5.92 (s, 1H), 3.07 (t, 2H), 2.27 (t, 3H), 1.61 (m, 2H), 1.26 (m, 29H), 0.88 (t, 3H) ppm


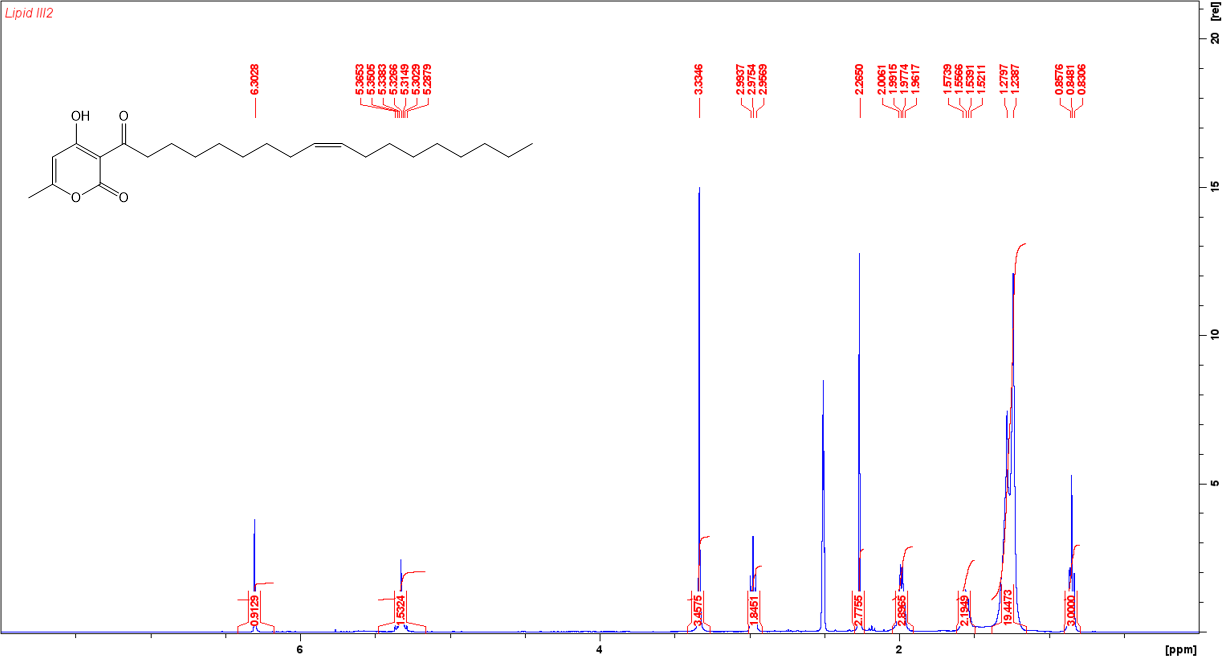


β, β’-triketone lipid III2 – Characterization Data: ^1^H NMR (400 MHz, DMSO-d_6_): δ 6.30 (s, 1H), 5.33 (m, 2H), 3.33 (s, 3H), 2.98 (m, 2H), 2.27 (s, 3H), 1.98 (m, 3H), 1.61 (m, 2H), 1.26 (m, 19H), 0.88 (t, 3H) ppm


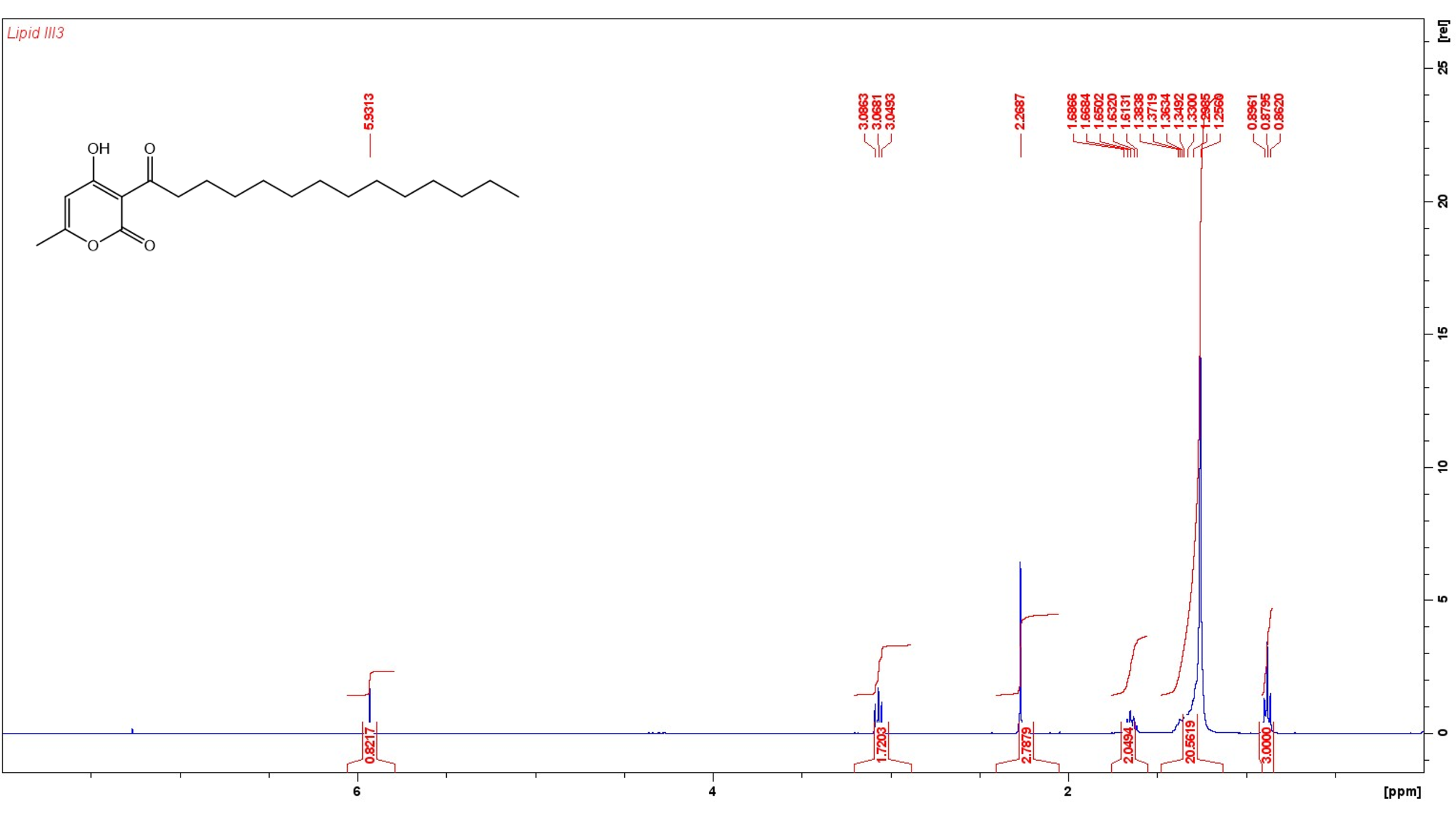


β, β’-triketone lipid III3 – Characterization Data: ^1^H NMR (400 MHz, CDCl_3_): δ 5.93 (s, 1H), 3.07 (t, 2H), 2.27 (t, 3H), 1.61 (m, 2H), 1.26 (m, 21H), 0.88 (t, 3H) ppm


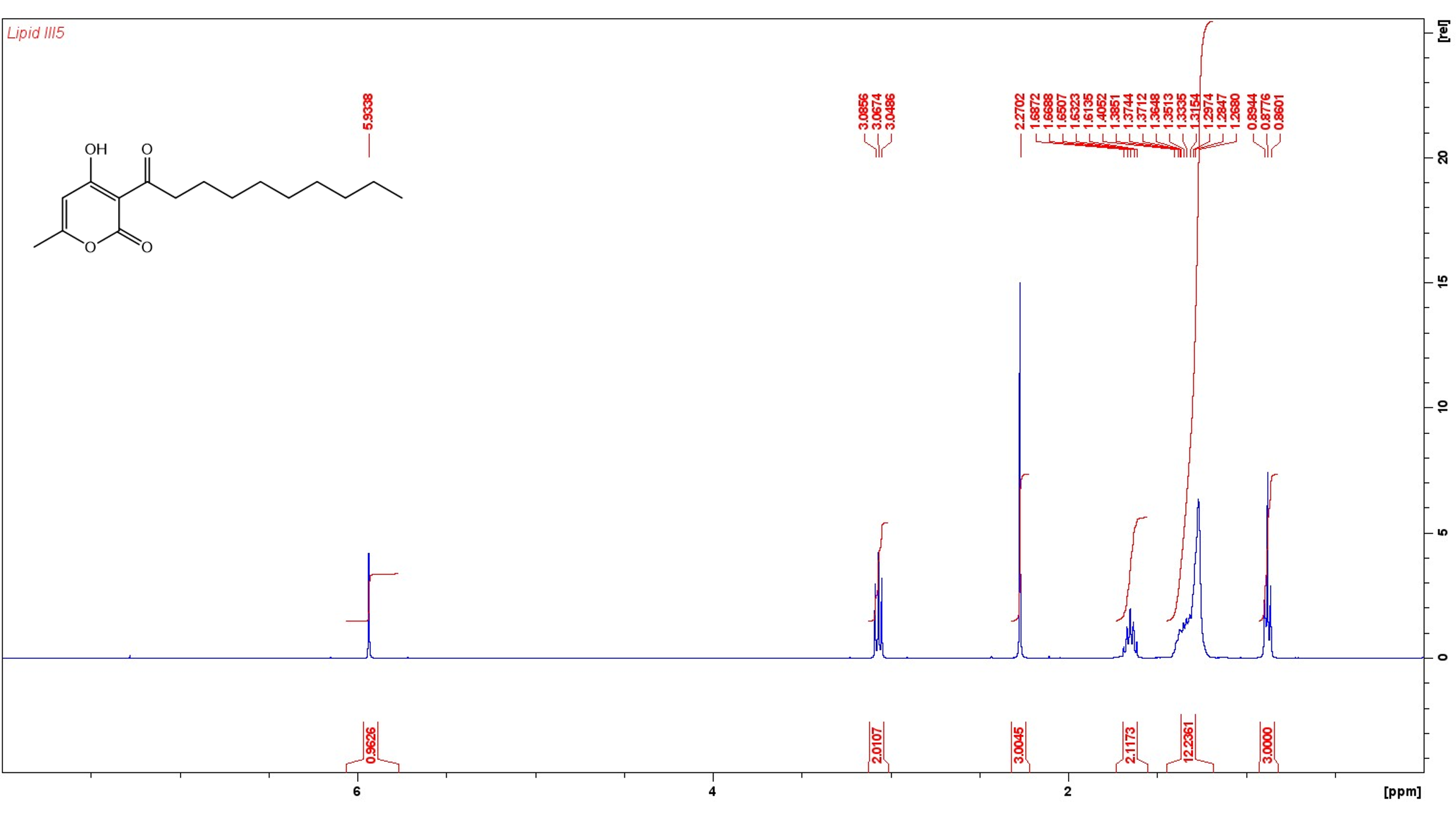


β, β’-triketone lipid III5 – Characterization Data: ^1^H NMR (400 MHz, CDCl_3_): δ 5.93 (s, 1H), 3.07 (t, 2H), 2.27 (t, 3H), 1.61 (m, 2H), 1.26 (m, 13H), 0.88 (t, 3H) ppm


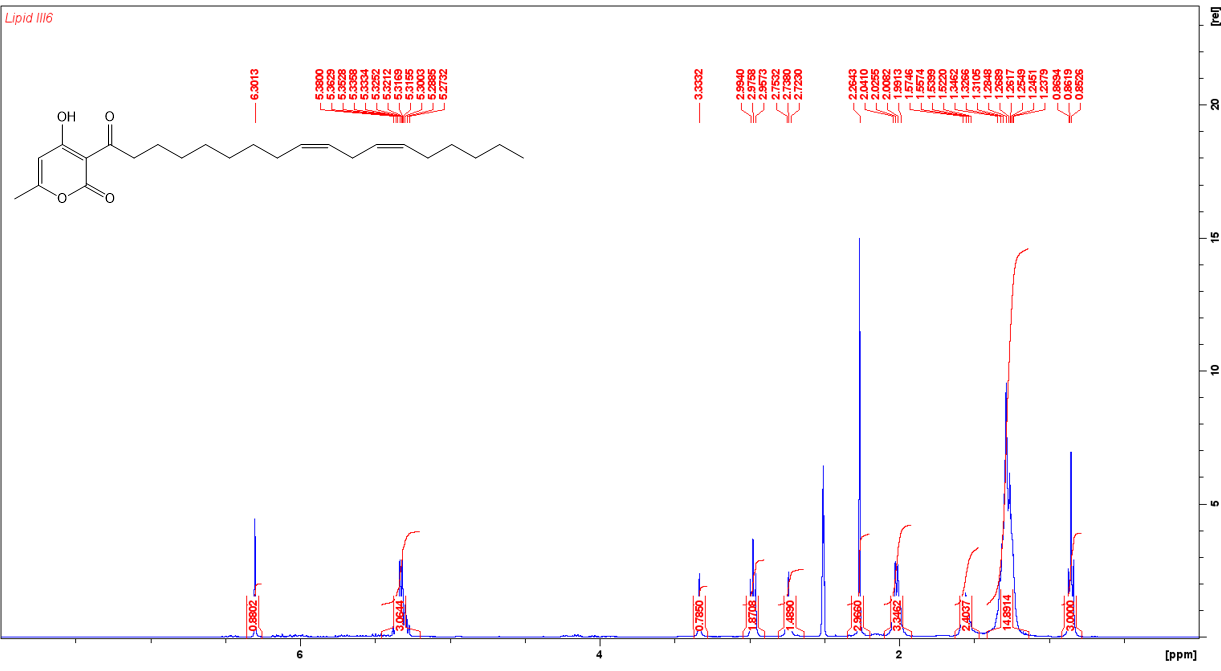


β, β’-triketone lipid III6 – Characterization Data: ^1^H NMR (400 MHz, DMSO-d_6_): δ 6.30 (s, 1H), 5.33 (m, 4H), 3.33 (s, 1H), 2.98 (t, 2H), 2.74 (t, 2H), 2.26 (s, 3H), 2.01 (q, 3H), 1.61 (m, 2H), 1.26 (m, 15H), 0.88 (t, 3H) ppm


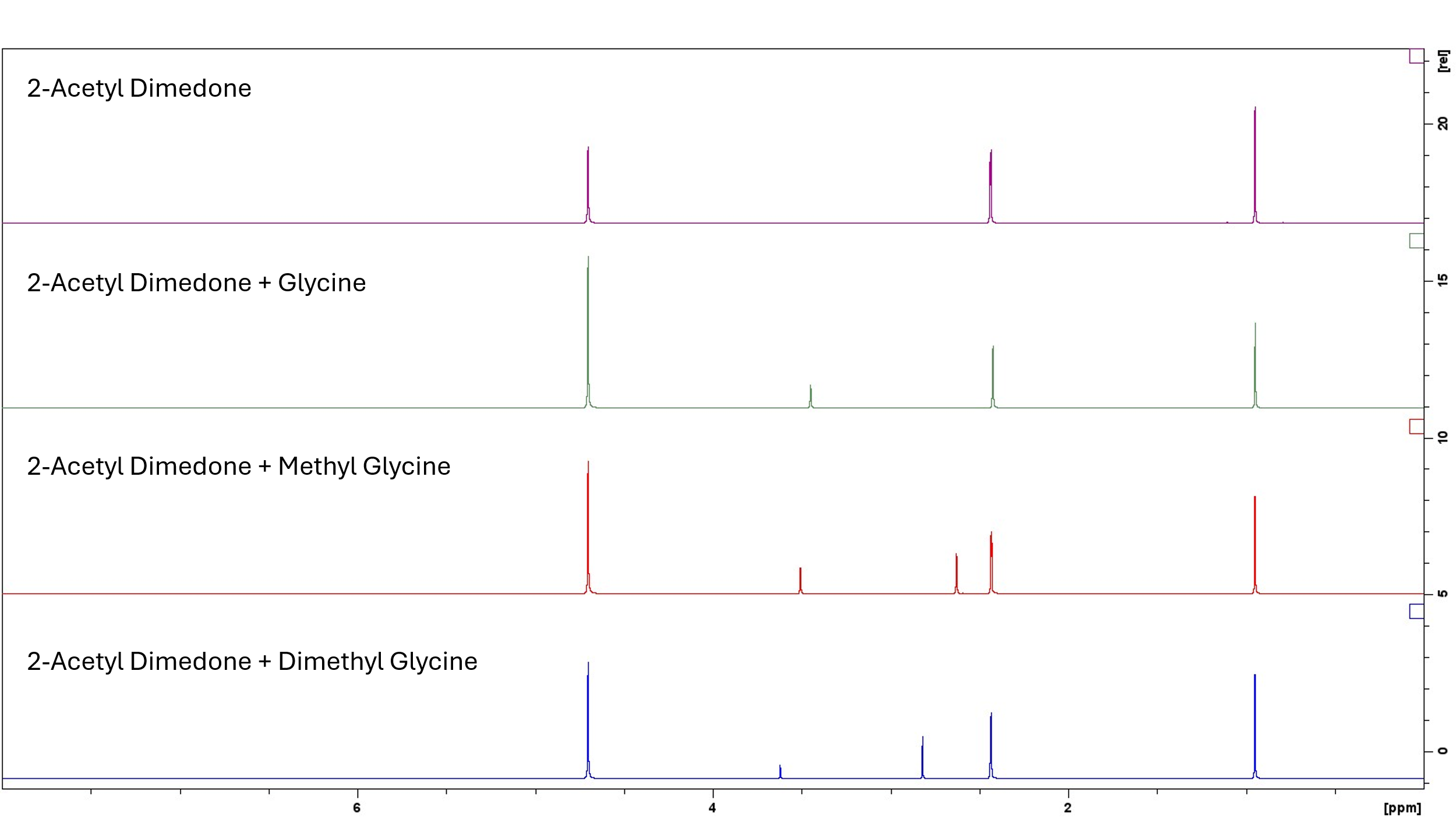


**Supplementary Figure 1**. Full ^1^H-NMR Spectrum of reaction products.


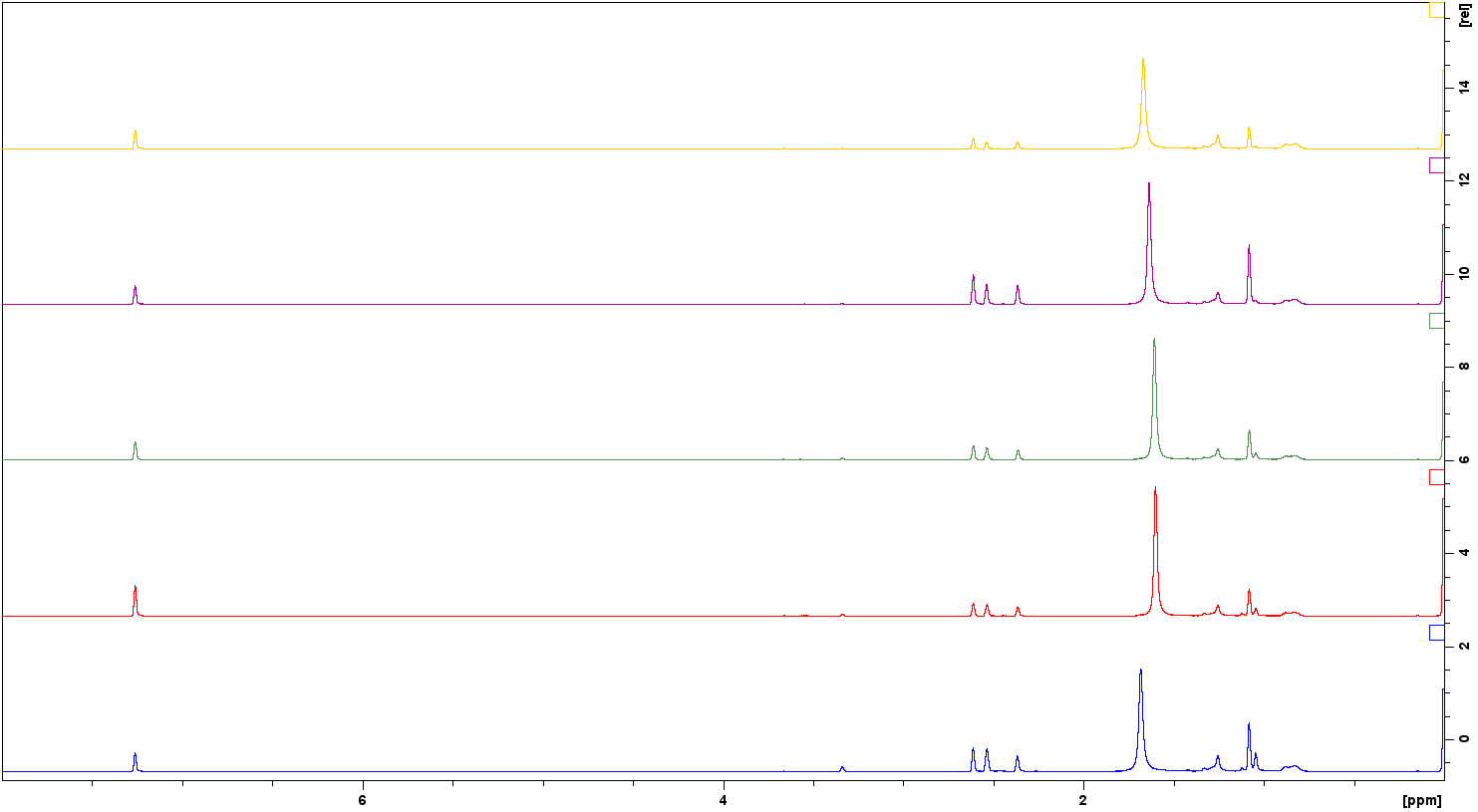


**Supplementary Figure 2**. Full ^1^H-NMR Spectrum of supernatant taken from the reaction mixture of 2-acetyl dimedone and glycine under pH 1 at 0 h, 1 h, 6 h, 12 h and 24 h (top to bottom)


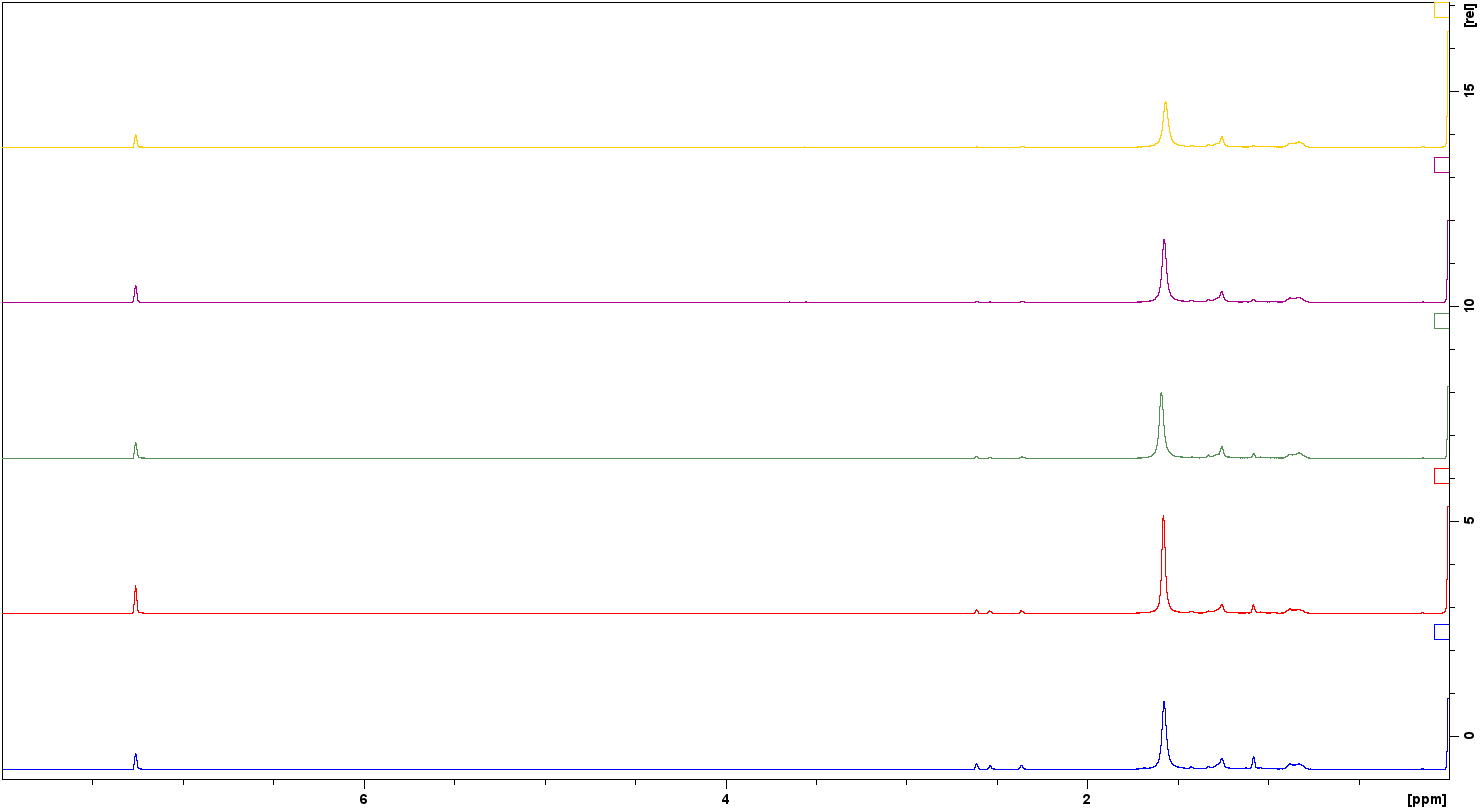


**Supplementary Figure 3**. Full ^1^H-NMR Spectrum of supernatant taken from the reaction mixture of 2-acetyl dimedone and glycine under pH 5.5 at 0 h, 1 h, 6 h, 12 h and 24 h (top to bottom)


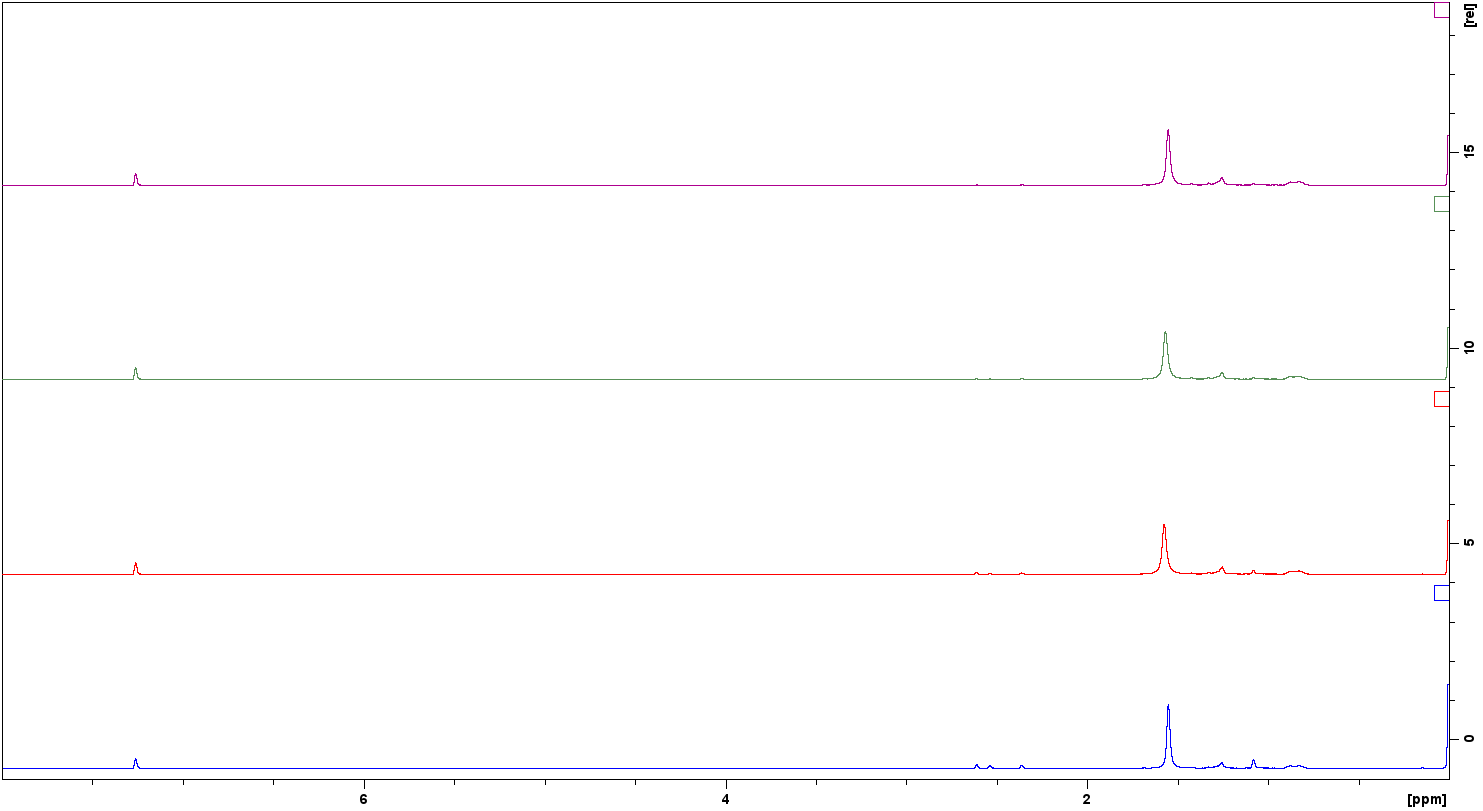


**Supplementary Figure 4**. Full ^1^H-NMR Spectrum of supernatant taken from the reaction mixture of 2-acetyl dimedone and glycine under pH 7.4 at 0 h, 1 h, 6 h and 24 h (top to bottom)

**Supplementary Figure 5**. Cytotoxicity of lipids.


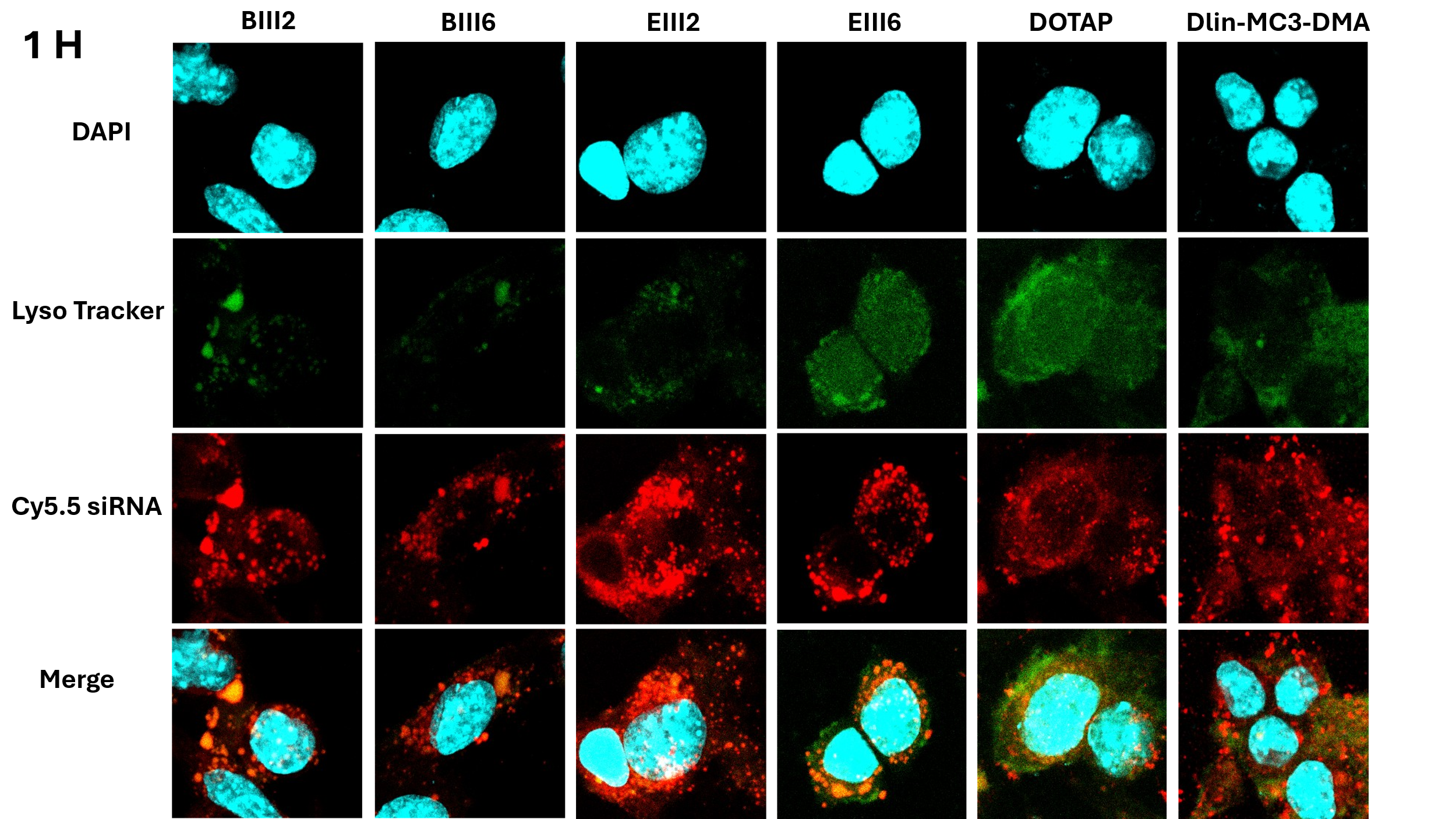


**Supplementary Figure 6**. Representative CLSM images of LNPs treated cells at 1 h post treatment.


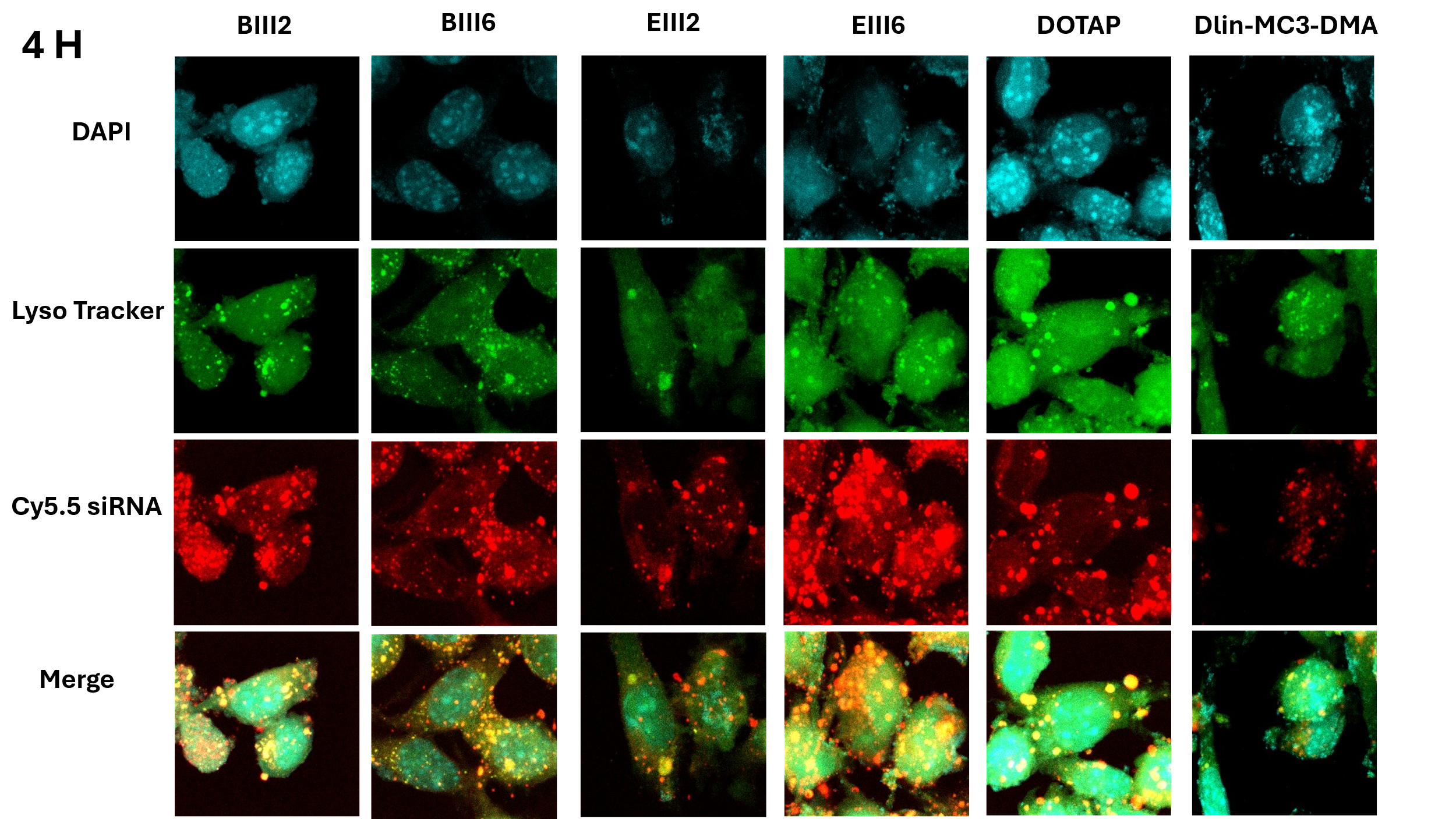


**Supplementary Figure 7**. Representative CLSM images of LNPs treated cells at 4 h post treatment.


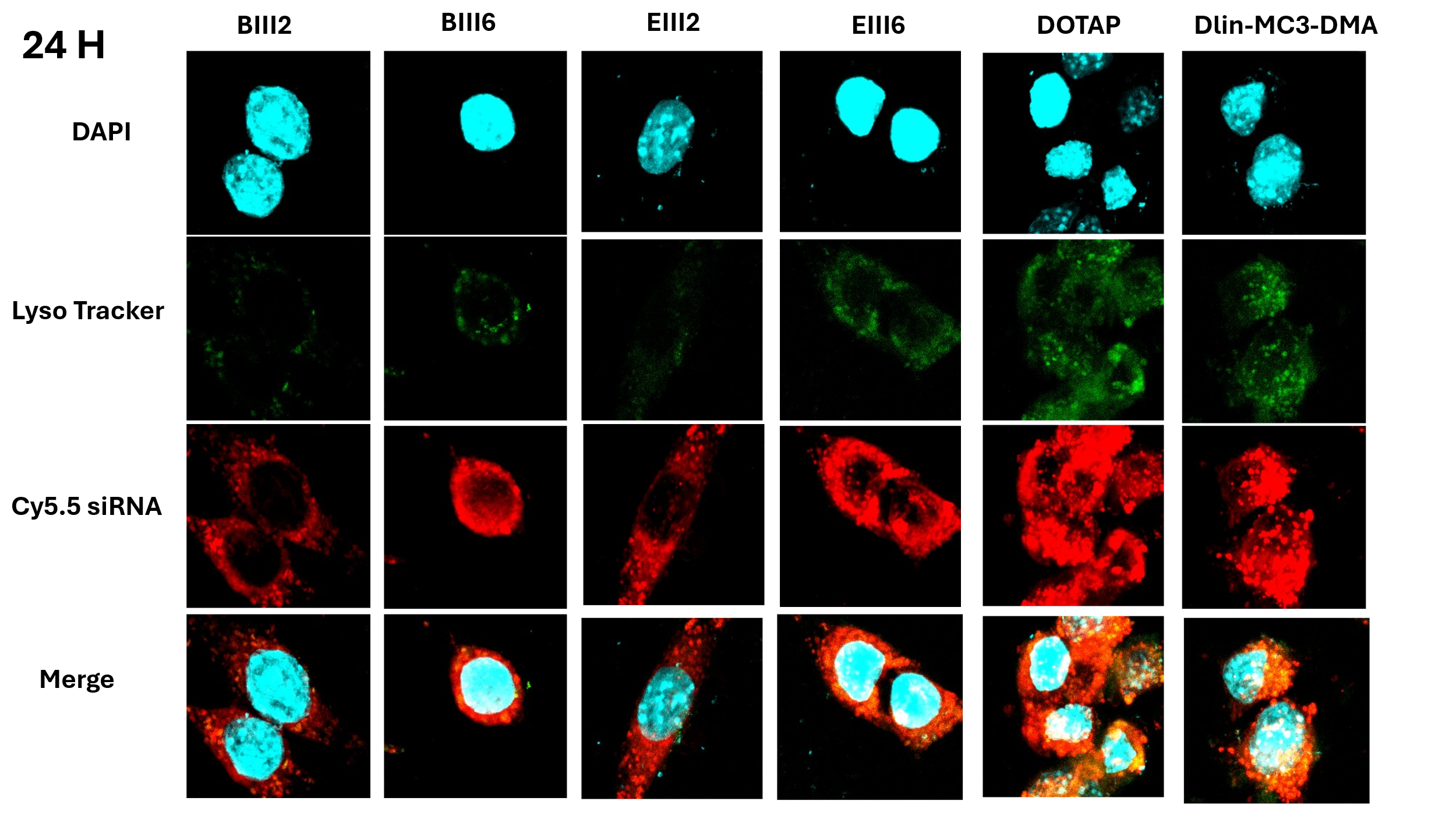


**Supplementary Figure 8**. Representative CLSM images of LNPs treated cells at 1 h post treatment.

**Supplementary Figure 9**. Change of body weight of mice receiving LNPs at different siRNA dosage in the dose-escalation toxicity study.


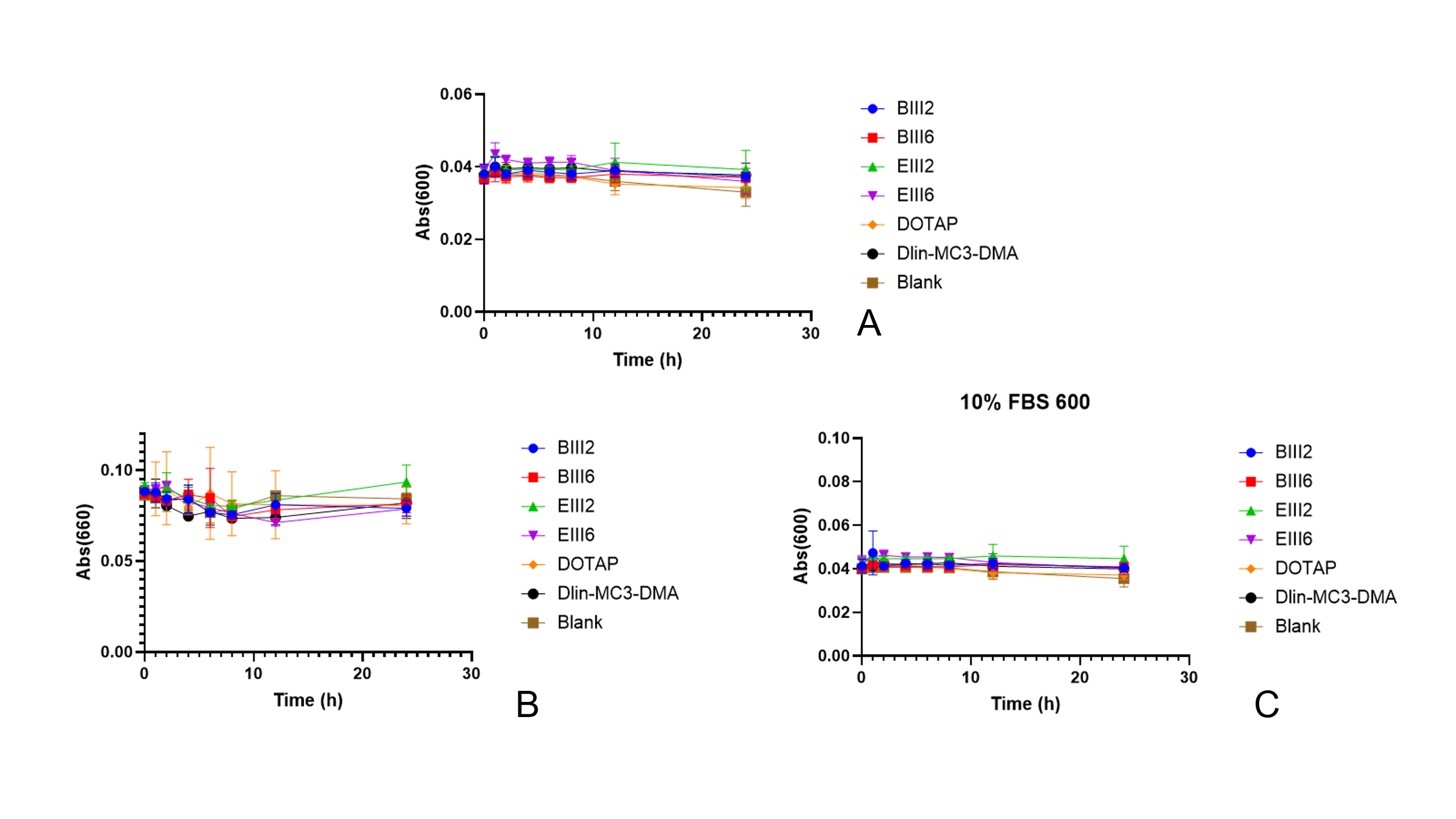


**Supplementary Figure 10**. Change of serum turbidity incubated with LNPs over time indicated by absorbance at 600 nm (**A**), and change of 10% FBS turbidity incubated with LNPs over time indicated by absorbance at 660 nm (**B**) and 600 nm (**C**).


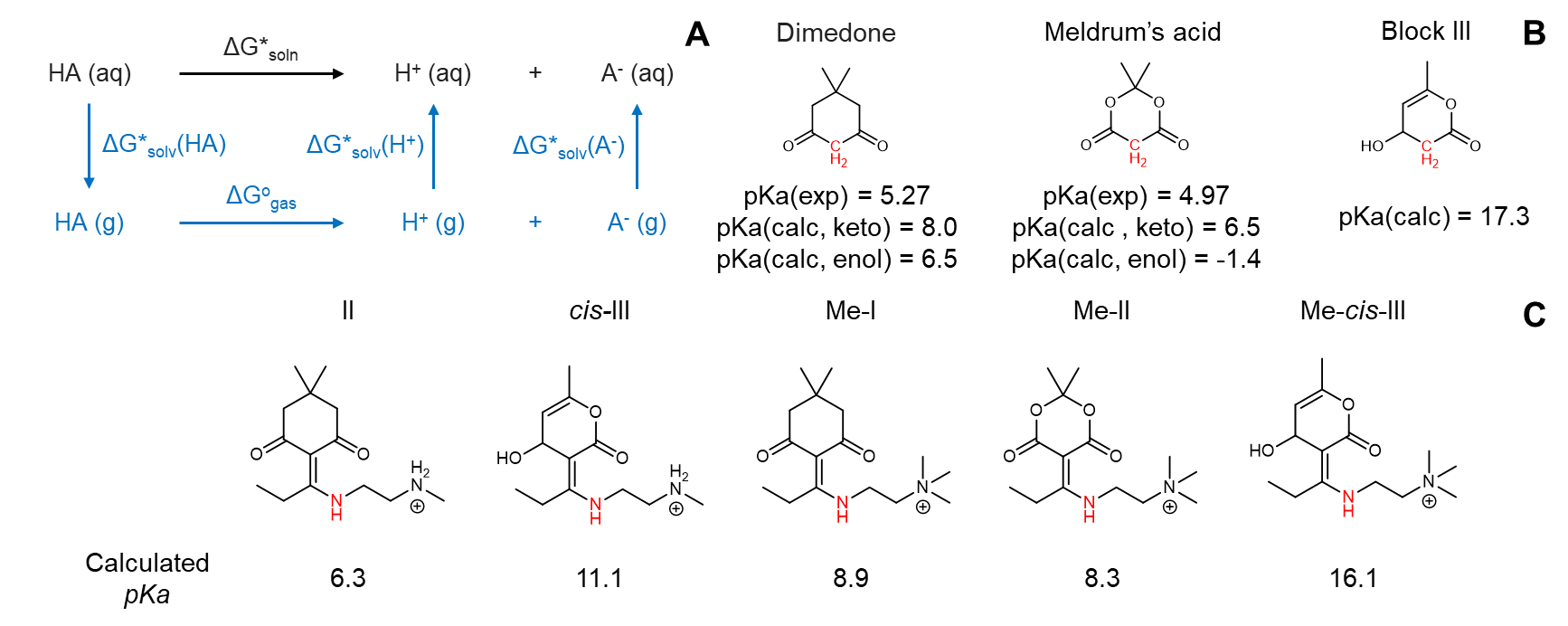


**Supplementary Figure 11**. pKa modelling of linkers. (**A)** The thermodynamic cycle used for pKa calculation. (**B)** Standard compound modeling. (**C)** Modeling and calculation of pKa of nitrogen H in Schiff base


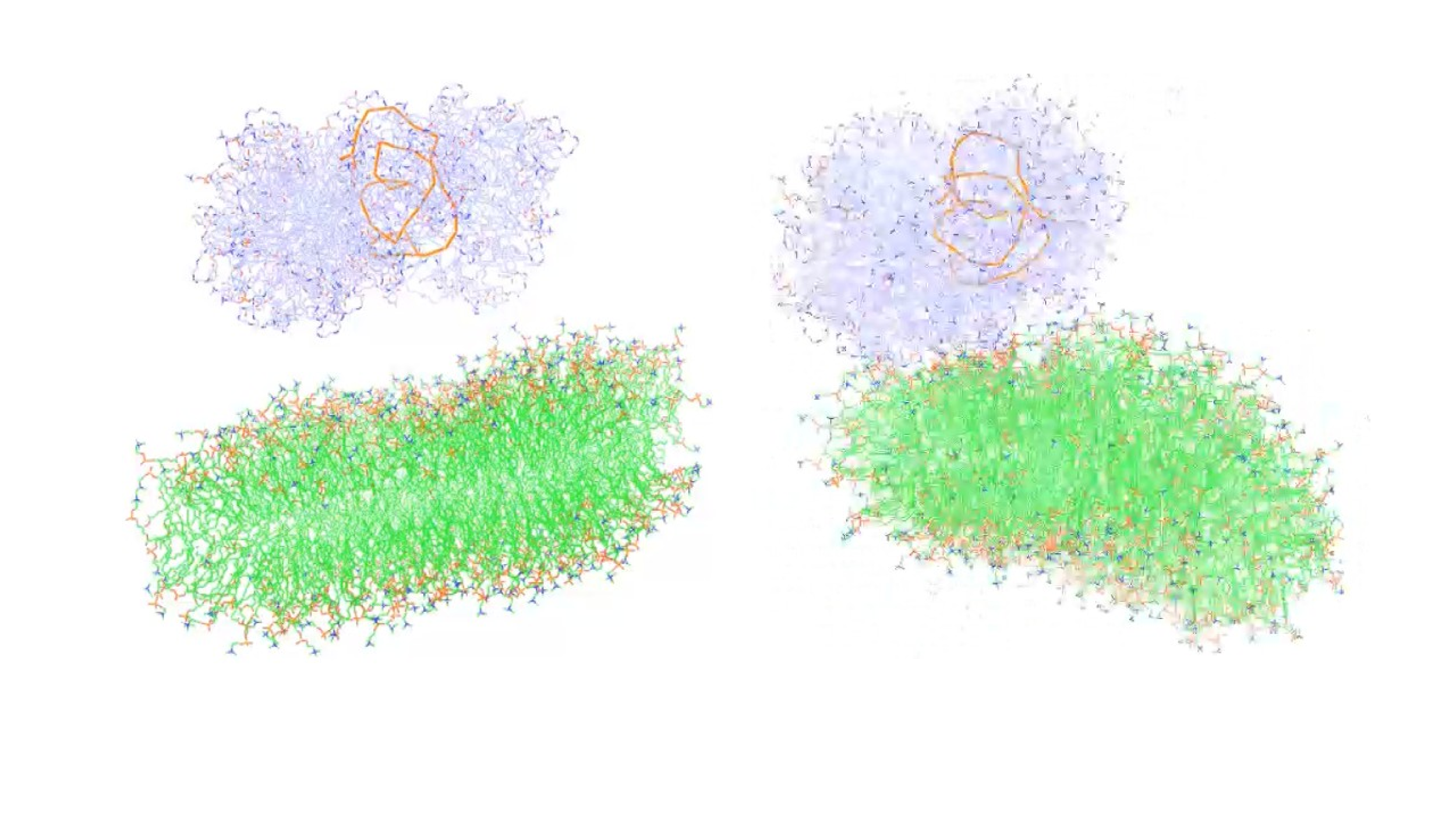


**Supplementary Figure 12**. Simulated process of siRNA-loaded BIII2 LNP fusing with a lipid bilayer. Left: Start; Right: End. Full simulation video attached separately to the manuscript.

**Supplementary Table 1: Characterization of selected LNPs**

| **LNP** | **Size (nm)** | **PDI** | **Zeta Potential (mV)** |
| --- | --- | --- | --- |
| BIII2 | 155.3 | 0.179 | -3.25 |
| BIII6 | 155.0 | 0.163 | -2.64 |
| EIII2 | 179.6 | 0.101 | 13.2 |
| EIII6 | 197.2 | 0.121 | 6.95 |
| Dlin-MC3-DMA | 123.1 | 0.338 | -3.98 |
